# New Insights into the LANCL2-**ABA** Binding Mode towards the Evaluation of New LANCL Agonists

**DOI:** 10.3390/pharmaceutics15122754

**Published:** 2023-12-12

**Authors:** Naomi Scarano, Francesco Di Palma, Nicola Origlia, Francesca Musumeci, Silvia Schenone, Sonia Spinelli, Mario Passalacqua, Elena Zocchi, Laura Sturla, Elena Cichero, Andrea Cavalli

**Affiliations:** 1 Department of Pharmacy, Section of Medicinal Chemistry, School of Medical and Pharmaceutical Sciences, University of Genova, Viale Benedetto XV, 3, 16132 Genoa, Italy; naomi.scarano@edu.unige.it (N.S.); francesca.musumeci@unige.it (F.M.); silvia.schenone@unige.it (S.S.); 2 Computational & Chemical Biology, Fondazione Istituto Italiano di Tecnologia, Via Morego 30, 16163 Genova, Italy; francesco.dipalma@iit.it (F.D.P.); andrea.cavalli@iit.it (A.C.); 3 National Research Council (CNR), Institute of Neuroscience, 56124 Pisa, Italy; nicola.origlia@in.cnr.it; 4 Laboratorio di Nefrologia Molecolare, IRCCS Istituto Giannina Gaslini, Via Gerolamo Gaslini 5, 16147 Genova, Italy; soniaspinelli@gaslini.org; 5 Department of Experimental Medicine, Section of Biochemistry, University of Genoa, Viale Benedetto XV 1, 16132 Genova, Italy; mario.passalacqua@unige.it (M.P.); ezocchi@unige.it (E.Z.); 6 Department of Pharmacy and Biotechnology, University of Bologna, Via Belmeloro 6, 40126 Bologna, Italy

**Keywords:** LANCL2, abscisic acid, molecular dynamics, AR-42, BT-11, mitochondrial proton gradient, virtual screening, agonist

## Abstract

The lanthionine synthetase C-like (LANCL) proteins include LANCL2, which is expressed in the central nervous system (CNS) and in peripheral tissues. LANCL2 exhibits glutathionylation activity and is involved in the neutralization of reactive electrophiles. Several studies explored LANCL2 activation as a validated pharmacological target for diabetes and inflammatory bowel disease. In this context, LANCL2 was found to bind the natural product abscisic acid (**ABA**), whose pre-clinical effectiveness in different inflammatory diseases was reported in the literature. More recently, LANCL2 attracted more attention as a valuable resource in the field of neurodegenerative disorders. **ABA** was found to regulate neuro-inflammation and synaptic plasticity to enhance learning and memory, exhibiting promising neuroprotective effects. Up until now, a limited number of LANCL2 ligands are known; among them, **BT-11** is the only compound patented and investigated for its anti-inflammatory properties. To guide the design of novel putative LANCL2 agonists, a computational study including molecular docking and long molecular dynamic (MD) simulations of both **ABA** and **BT-11** was carried out. The results pointed out the main LANCL2 ligand chemical features towards the following virtual screening of a novel putative LANCL2 agonist (**AR-42**). Biochemical assays on rat H9c2 cardiomyocytes showed a similar, LANCL2-mediated stimulation by **BT-11** and by **AR-42** of the mitochondrial proton gradient and of the transcriptional activation of the AMPK/PGC-1α/Sirt1 axis, the master regulator of mitochondrial function, effects that are previously observed with **ABA**. These results may allow the development of LANCL2 agonists for the treatment of mitochondrial dysfunction, a common feature of chronic and degenerative diseases.

## 1. Introduction

The lanthionine synthetase C-like (LANCL) enzyme family is named after its sequence homology with the bacterial lanthionine synthetase, producing lantibiotics as natural antibiotics from modified cysteine. In particular, the prokaryotic LANCLs represent zinc-containing enzymes involved in the modification and transport of peptides and lanthionines [1]. Mammalian LANCL proteins consist of LANCL1, LANCL2, and LANCL3 enzymes. LANCL3 exhibits the lowest expression levels among these LANCL proteins and may even be a pseudogene [2,3].

LANCL1 was the first member of this family to be isolated from human erythrocyte membranes [4] and then to be highly expressed in the brain [1,5], while LANCL2 was subsequently recognized as expressed at the central nervous system (CNS) level, as well as in peripheral tissues, such as the immune cells, heart, placenta, lung, liver, pancreas, prostate, and skeletal muscles [6]. Furthermore, LANCL1 and LANCL2 differ in their sub-cellular localization: LANCL1 is mainly a cytosolic and nuclear protein, while the majority of LANCL2 is inserted into the plasma membrane by means of the myristoylated Gly residue at the N-terminal of the protein [7]. 

**Figure 1:**
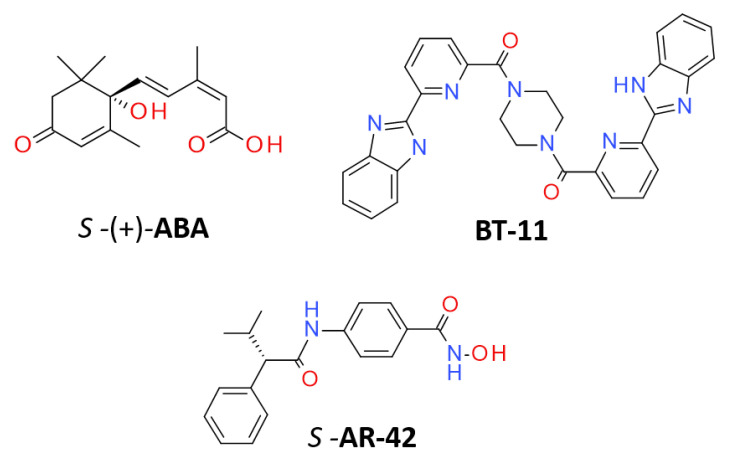
Chemical structure of the known LANCL2 agonists (**ABA** and **BT-11**) and of the in silico screened **AR-42**.

Both LANCL1/2 have been demonstrated to bind the natural product abscisic acid (**ABA**) [8], a plant hormone also produced and active in animals; it exists in two enantiomers: *S-*(+)-**ABA**, which is the most abundant form in plants, and *R*-(−)-**ABA** (Figure 1).

**ABA** is an isoprenoid phytohormone that regulates the response to abiotic stress (temperature, light, or drought), seed dormancy, and germination in plants [9,10], being able to act as a hormone in mammals. Several studies suggested **ABA** modulation in the release of insulin from β-pancreatic cells and in the activity of immune cells [11,12]. In particular, **ABA** stimulates insulin release from human and murine β-pancreatic cells, via a PKA-dependent activation of CD38. In addition, it has been proven that **ABA** binding and signaling in human granulocytes caused the activation of adenylate cyclase (AC), the cAMP-dependent activation of protein kinase A (PKA), the phosphorylation of the cADPR CD38, and the related cADPR overproduction, causing an increased level of intracellular Ca^2+^ concentration [11,12,13,14]. Recently, **ABA** and its mammalian receptors LANCL1 and LANCL2 have been shown to play an important role in muscle cells and cardiomyocytes, improving glucose uptake, the cell cycle and vitality, cytoskeletal and contractile protein synthesis, and mitochondrial functions with an increased mitochondrial proton gradient (ΔΨ), mitochondrial DNA content, and respiration via a signaling pathway involving the AMPK/PGC-1α/Sirt1 axis [8,15,16]. 

In agreement with these patterns of activity profiles, **ABA** exhibited pre-clinical effectiveness in the treatment of diabetes and other inflammatory diseases such as atherosclerosis [17,18,19,20,21]. Accordingly, LANCL2 was, so far, investigated as a promising target for chronic inflammatory, metabolic, and immune-mediated diseases, such as inflammatory bowel disease (IBD), and cancer [21,22]. 

Interestingly, more recent studies regarding **ABA** in the central nervous system (CNS) have found that it can reduce neuroinflammation, aiding in synaptic plasticity to enhance learning and memory [23,24]. Experiments suggested that **ABA** administration can prevent memory dysfunction, while more recent analyses investigated the therapeutic potential of **ABA** and related mechanisms in the pathology of Alzheimer’s disease (AD) and neurodegenerative disorders [23].

Up until now, only a limited number of **ABA** analogues was biologically assessed and investigated as LANCL2 ligands, as well as other structurally different chemical entities [25,26]. Only one patented compound, namely, **BT-11** (see Figure 1) [27], has been described as an LANCL2 agonist able to reduce inflammation in multiple mouse models of IBD (Omilancor, Phase 2 recently completed) [28], also with lead indications in ulcerative colitis (UC) and Crohn’s disease (CD) [29]. **BT-11** was tested for general toxicity in studies in rats and dogs, revealing the compound to be a safe and well-tolerated oral drug candidate with no observed adverse effect at a level > 1000 mg/kg in in vivo studies [29].

On this basis, the design and biological evaluation of new drug-like LANCL2 agonists could represent a promising approach for the development of neuroprotective and anti-inflammatory agents.

Our previous studies allowed us to explore in silico different **ABA** binding sites in the developed hLANCL2 homology model [30]. The most probable (Site 1) was delimited by LEU111, LEU114, ARG118, GLN115, TYR205, TYR209, THR212, and GLU213. Furthermore, other putative **ABA** high-affinity binding sites (Site 2 and Site 3) were probed. These theoretical data have been validated as a result of the site-directed mutagenesis of Site 1, with and without the concomitant mutation in either one of the other sites (Site 2 or Site 3), to repeat the binding experiments on the mutated proteins [30]. 

The very recently released X-ray crystallographic data of the human (h) LANCL2 protein (PDB code = 6WQ1) [2] offer the unprecedented opportunity to evaluate in silico new ligands via future structure-based virtual screening (VS). 

Here, we developed for the first time a long molecular dynamic (MD) simulation of the complete hLANCL2 model as derived by the aforementioned new PDB code 6WQ1. Then, we exploited this structural information to elucidate the binding pose of (*R/S*)-**ABA** in its high affinity binding site (Site 1) via molecular docking, followed by long MDs. The derived complexes were simulated for 1 μs by means of all-atom molecular dynamics (MDs), producing five replicas for each system. Similarly, the known drug-like LANCL2 agonist **BT-11** was also investigated in silico via the previously cited PDB code 6WQ1, to clarify the most relevant LANCL2 agonist interaction pattern. The results obtained allowed us to point out the main features supporting the compounds’ ability to target LANCL2 and to proceed with the subsequent evaluation of a novel promising LANCL2 agonist (**AR-42**) (see Figure 1). This compound has been previously reported as a histone deacetylase (HDAC) inhibitor for cancer treatment [31].

With the aim of supporting these novel computational investigations in the search of new putative LANCL2 ligands, we also reported herein biochemical assays concerning **ABA** and **BT-11** as reference LANCL2 agonists, as well as for the newly investigated derivative (**AR-42**). The experimental studies have been added to give a first validation of the newly proposed LANCL2 agonist **AR-42**, by comparison with the known compounds **BT-11** and **ABA**. In detail, mitochondrial function analyses in LANCL2-overexpressing and LANCL2-silenced cardiomyocytes cells have been performed, when treated with the mentioned ligands, using the mitochondrial proton gradient (∆Ψ)-sensitive dye JC-1 [5,32]. Indeed, mitochondrial dysfunctions, such as excessive reactive oxygen species (ROS) production, mitochondrial Ca^2+^ dyshomeostasis, the loss of ATP, and defects in mitochondrial dynamics and transport and mitophagy, are expected to have a high impact on the pathogenesis of neurodegenerative diseases such as AD [33,34]. In addition, an expression analysis of genes involved in neuroprotection and anti-inflammatory events, such as AMPK, PGC-1α, and eNOS [35,36], was performed by qPCR on LANCL2-overexpressing and LANCL2-silenced cardiomyocytes H9c2 cells, treated with **ABA**, **BT-11**, and **AR-42**. 

The results obtained encourage further deepening efforts to definitively validate hLANCL2 as a viable therapeutic target for the treatment of neurodegenerative disorders and the discovery of novel promising neuroprotective/anti-oxidant/anti-inflammatory agents.

## 2. Materials and Methods

### 2.1. Computational Studies

#### 2.1.1. Modelling of the Missing LANCL2 Residues

The X-ray structure of LANCL2 (PDB code = 6WQ1) [2] was missing residues 1–21 (N-terminus), 445–450 (C-terminus), and 34–60 (internal loop). The unsolved portions were modelled using two alternative methodologies, namely, homology modeling generation by means of MOE [37], and ab initio prediction using the artificial intelligence system AlphaFold [38,39], as AF models. In more detail, the model predicted by Alphafold2 [35] was retrieved as stored in the Alphafold Protein Structure Database [39,40].

The MOE Homology Model (HM) was built similarly to a previously reported method [30]. Briefly, the HM was generated using as reference the available X-ray structure of LANCL2 (PDB code: 6WQ1; resolution: 2.295 Å) [2]. The protein sequence was retrieved from the protein data bank itself, and the alignment was performed according to the Blosum62 matrix (MOE software-2019.01 version). One hundred models were produced using a Boltzmann-weighted randomized procedure, and loop building was performed according to a knowledge-based procedure [37]. The results were ordered according to packing score, a function derived from a statistical analysis of residues’ typical environment. The obtained models were visually inspected, and after duplicate (or close analogue) removal and exclusion of unfeasible conformations, the three top-scored models were retained. These models were submitted to 1000 minimization steps with the steepest descent algorithm, and the minimized structures were used for pocket calculation with both MOE SiteFinder and the Pocketron module by BikiLifeScience [41]. The purpose of the pocket calculations was to assess if one of the models would be able to host the endogenous activator in the previously reported Site 3, as the modelled loop (3–60) was reported to weakly bind **ABA** [30]. In this regard, it would be worth mentioning that the reconstructed loop is the distinguishing feature between LANCL2 and LANCL1 amino acid sequence, with the latter one lacking the whole loop (see Figure S1).

Then, the top-scored model (HM1) was kept for further studies.

Apart from the reconstructed loop subdomain, some considerations on both terminals were made. Terminal modelling was of utmost importance for LANCL2 system, as **ABA** binding occurs very closely to the C-terminus. For these regions, the most folded/stabilized conformations available were selected, with the C-terminus conformations that “close” the **ABA** site retained as preferred. In addition to MOE software, an ab initio modelling of the full-length protein was also attempted with Alphafold2.0 [38] as previously mentioned. The differently modelled gaps and terminals were manually integrated in the original X-ray (6WQ1) pdb file [2].

#### 2.1.2. Molecular Docking Studies

The ligand molecules were built through MOE Builder and imported in Maestro [42]. The LigPrep module was utilized for ligand preparation, assigning the prevalent protonation and tautomeric state. The previously obtained models for LANCL2 protein were prepared using the Protein Preparation Wizard. After water elimination, the hydrogens were assigned to the protein optimizing the H-bond networks. The docking grid center was set as the centroid of GLN115, GLU213, HIS426, PRO440, and LEU444 residues, and the size of the box of interest was enabled to dock ligands with length **≤** to 20 Å. The scaling factors were kept at default values. The docking protocol followed a three-step procedure using Maestro [42]. The three steps comprised an SP (standard precision), an XP (extra precision), and an induced-fit docking step. Briefly, the SP docking protocol involves a series of hierarchical evaluations to select the best docking poses. An extensive conformational search generates a set of ligand conformations, and the spatial compatibility between the ligand conformation and the protein is assessed. A grid representation of the target involving several fields is used to this aim. The poses selected from these filters are submitted to ligand minimization in the target field using the OPLS3 force field (FF) [43]. The lowest-energy poses are then submitted to Monte Carlo simulations, to optimize the tortional energy and ranked according to the default scoring function (GlideScore) [44,45,46]. The XP protocol follows an anchor-and-grow sampling approach and a stricter scoring function with respect to SP docking [44,47]. The induced fit (IF) procedure (IFD) comprehends the docking of the ligand with Glide, with reduced van der Waals radii and increased non-bonded energy interactions cutoff. Highly flexible side chains may be removed during this initial modified docking. For each of the obtained poses, the Prime module performs a prediction step on the surrounding amino acids to allow ligand accommodation by re-orienting residue side chains. Both these residues and the ligand are then minimized and each ligand was re-docked in its specific binding site conformation [44,48,49]. The results, including five docking poses, were ordered according to the docking score/Glide GScore (scoring function in the case of SP and XP, and IFD score in the case of IF). Considering the IF results (see Tables S1–S4), the best poses were chosen based on the following criteria: (i) The external part of the site is rich in positively charged residues (arginines, lysines, etc.), while the internal part is less charged. Therefore, the **ABA** COO- would be more likely to interact with the positively charged area. Moreover, the poses in which this rule is observed were the majority and generally top-scored with respect to the “inverted” ones (COO- towards the internal part of the protein). (ii) A key polar contact has to be established with the mutated R118, (iii) number of detected H-bonds, and (iv) interaction conservation passing from *R-* to *S-***ABA** (as both the enantiomers were shown to be active).

Thus, four complexes were obtained: *R-***ABA**/LANCL2 (model AF), *R-***ABA**/LANCL2 (model MOE), *S-***ABA**/LANCL2 (model AF), and *S-***ABA**/LANCL2 (model MOE).

For the docking of **BT-11**, we followed an identical protocol (three-step docking involving an induced fit procedure), using the holo-form of the AF-completed LANCL2 structure (see Table S5). This choice was derived by the analysis of the **ABA**/LANCL2 dynamics, for which the AF-completed model exhibited a more stable binding with the ligand. No MD steps were made before docking. After calculation, we selected three poses from the IF output (see **BT-11**:LANCL2 MD simulations section of the Results). VS via molecular docking of **AR-42** was performed, applying the aforementioned docking protocol, focusing on the LANCL2 site 1.

#### 2.1.3. MD Simulations

The obtained complexes *R-***ABA**:LANCL2(MOE) and *S-***ABA**:LANCL2(MOE), *R-***ABA**:LANCL2(AF) and *S-***ABA**:LANCL2(AF), and **BT-11**:LANCL2 (hybrid:terminals from AF, loop from MOE) and apo-structures were submitted to MD with the GROMACS 2020 engine [50]. Recently developed FF parameters for zinc and zinc-chelating residues [51,52] were implemented in the amber14sb FF [53]. Small molecules were parametrized with AmberTools22 [54], using GAFF [55]. Charge calculation was performed according to the AM1-BCC method [56,57]. A dodecahedron-shaped box was built and solvated using the TIP3P explicit model for water [58]. A salt concentration (NaCl) of 0.15 M was imposed in addition to electro-neutrality requirement to reproduce the experimental environment of **ABA** binding experiments [30]. Energy minimization was carried out with the steepest descent algorithm and terminated as the maximum force decreased to less than 1000 kJ mol^−1^ nm^−1^. Three equilibrations were performed as reported in Table S6. In NPT conditions, the Berendsen barostat [59] was used to keep pressure constant.

Finally, 1 μs production simulations were carried out with the leapfrog algorithm and a time step of 2 fs. During the production step, the simulation temperature was set to 300 K, and pressure was kept constant with the Parrinello–Rahman barostat [60]. Five replicas of 1 μs were produced for each of the investigated systems. Simulations of LANCL2 in the apo-form were run as control. Calculations were run on an NVIDIA TESLA V100 multi-GPU cluster. VMD [61] and PyMOL [62] were used to visualize the trajectories and investigate the key interactions between the protein and the ligand, respectively. XMGrace [63] was used to produce RMSD and RMSF plots. H-bond analysis results were analyzed by means of the readHBmap.py script [64]. For contact analysis, the calculation was run with GROMACS [50] hbond module, using the -contact flag. The distance from the ligand was set at 3 Å. The results were analyzed via an awk/python script. Contacts in which only one atom of the protein was within the 3 Å threshold were excluded. Visual investigation on the dynamics (VMD) [61] was performed to verify the putatively individuated non-polar interactions.

### 2.2. Chemical Structures

The molecules *S-*(+)-**ABA**, **BT-11**, and *S-***AR-42** were purchased from Merck (Milan, Italy).

### 2.3. Biochemical Assays

#### 2.3.1. Cell Culture

Rat embryonic cardiomyocyte H9c2 cell line was purchased from ATCC (LGC Standards s.r.l., Milan, Italy) and was cultured in DMEM high glucose (Sigma-Aldrich, Milan, Italy) supplemented with 10% fetal bovine serum (Sigma-Aldrich, Milan, Italy), penicillin (62.5 μg/mL), and streptomycin (100 μg/mL) (Sigma-Aldrich, Milan, Italy). Cells were kept at 37 °C in a humidified atmosphere with 5% CO_2_. Overexpression (OVL2) or silencing (SHL2) of LANCL2 were obtained using retroviral or lentiviral infection, respectively, as described before [8,15]. Briefly, overexpression of LANCL2 (OVL2) was obtained in rat H9c2 cells using retroviral pBABE vectors constructed as described in [8], using the empty vector pBABE (Addgene) as negative control. The lentiviral plasmids pLV[shRNA]-Puro-U6 encoding for a control scramble shRNA (SCR) and for shRNA targeting rat LANCL2 (SHL2) (plasmid ID: VB010000-0005mme and VB181016-1124zjp) were purchased from Vector Builder (Chicago, IL, USA). Retroviral and lentiviral transductions were performed as described in [8]. LANCL2 overexpression and silencing were confirmed by qPCR and by Western blot experiments as described before [8].

#### 2.3.2. Cell Vitality Assay

H9c2 cells were seeded into 96-well plates at a density of 5 × 103 cells/well and cultured for 24 h. Then, cells were incubated with 1, 5, 10, 50, and 100 µM **BT-11** and **AR-42**, respectively (100 μL/well). After 24 h of incubation, cell vitality was assessed by resazurin (7-Hydroxy-3H-phenoxazin-3-one 10-oxide) assay [65], adding, at 15 μL/well, a 0.015% solution of resazurin in PBS and incubating for 4 h. The percentage of viable cells was determined measuring fluorescence with filter set Ex544/Em590 in a microplate reader (CLARIOstarPlus BMG Labtech, Ortenberg, Germany).

#### 2.3.3. JC-1 Analysis

OVL2 and SHL2 H9c2 cardiomyocytes were stained with the cationic dye JC-1 (ThermoFisher Scientific, Waltham, MA, USA), which exhibits potential-dependent accumulation in mitochondria. At low membrane potentials, JC-1 exists as a monomer and produces a green fluorescence (emission at 527 nm), whereas, at high membrane potentials, JC-1 forms aggregates and produces a red fluorescence (emission at 590 nm). Thus, mitochondrial depolarization is indicated by a decrease in the red/green fluorescence intensity ratio [32]. Cells were treated with the same method already described in [15]. Briefly, H9c2 cells were seeded at 2 × 10^4^ onto µ-slide wells, treated or not with 10 µM **ABA**, **BT-11**, and **AR-42** for 2 h, stained with JC-1 (2.5 µg/mL) for 20 min at 37 °C in a 5% CO_2_ incubator, and then imaged live. The red/green ratio was analyzed after a background subtraction with the ImageJ v1.8.0 software [66] using a quantitative analysis based on an intensity measurement of specific selected ROIs.

#### 2.3.4. qPCR Analysis

OVL2 and SHL2 H9c2 cells were incubated with or without 10 µM **ABA**, **BT-11**, and **AR-42** for 4 h after being serum-starved for 18 h. Total RNA was extracted from cardiomyocytes using RNeasy Micro Kit (Qiagen, Milan, Italy), according to the manufacturer’s instructions. cDNA was obtained from 1 μg of total RNA by using iScript cDNA Synthesis Kit (Bio-Rad, Milan, Italy) and qPCR reactions were performed in an iQ5 Real-Time PCR detection system (Bio-Rad, Milan, Italy) as described in [4]. Specific rat primers were designed using Beacon Designer 2.0 software (Bio-Rad, Milan, Italy), and their sequences are listed in Table S7.

Values were normalized on hypoxanthine-guanine phosphoribosyltransferase-1 (Hprt1) mRNA expression. Statistical analysis of the qPCR was performed using the iQ5 Optical System Software version 1.0 [Bio-Rad Laboratories, Milan, Italy] by 2^−△△Ct^ method [8]. The dissociation curve for each cycle of amplification was analyzed to confirm the absence of nonspecific PCR products.

## 3. Results

Recently, the X-ray structure of apo-LANCL2 (6WQ1) was published [2]. This piece of information paved the way for the development of structure-based studies devoted to the design of novel putative ligands. On this basis, we proceeded with molecular docking studies followed by MD simulations of the known endogenous ligand **ABA** at LANCL2.

Initially, we modelled gaps of the crystal structure of LANCL2 with AlphaFold [38,39] and MOE [34] (see Section 2), obtaining MODEL1 and MODEL2, respectively. These two alternatives were used to perform the molecular docking of *R/S*-**ABA** in LANCL2 Site 1, as we have previously described via mutagenesis experiments [30].

Details of the conformer scoring functions as obtained for the best five ranked poses are shown in Tables S1–S4. These steps were performed prior to any MD simulations.

Afterwards, 1 μs MDs of the mentioned systems were carried out to in silico validate the mode of interaction of the LANCL2 agonist with the target, leading to a comparable **ABA** binding mode. Then, another known LANCL2 activator was considered (**BT-11**) via molecular docking and MD simulations. In particular, we speculated that this synthetic activator may bind at the same binding site as **ABA** to exert LANCL2 activation. We therefore applied a three-step (standard precision—SP, extra precision—XP, and induced fit—IF) docking protocol to **BT-11** (see Table S5), and then we selected three poses for MD validation (see Section 2).

The two top-scoring poses were considered, as well as pose 5. The latter was chosen because this pose exhibits a quite different positioning if compared to poses 1–2 than the other poses 3 and 4.

The aforementioned apo-LANCL2, as well as holo-LANCL2 simulations, are described hereafter.

### 3.1. Computational Studies

#### 3.1.1. Apo-LANCL2 MD Simulations

At first, to proceed with the apo-LANCL2 MD simulation, modelling of the missing residues at the available X-ray LANCL2 structure has been performed. The completion of the LANCL2 crystal structure (PDB code = 6WQ1) [2] was performed with two different methods—(i) via AlphaFold (MODEL1) [38,39] and (ii) referring to MOE-generated homology models (HMs; MODEL2)—starting from the just-mentioned 6WQ1-deposited structure [2]. The obtained models (MODEL1 and MODEL2) exhibited a good stabilization of the C- and N-terminus and were jointly considered for further studies (see experimental section for details). The experimental data concerning 6WQ1 exhibit compact conformation and a stabilized conformation of the C- and N-terminus. The completed structures with the two methods are reported in Figure S2.

The two selected models were submitted to the 1 µs MD, and the corresponding trajectories were analyzed as follows to verify the simulation stability and gain information on the flexibility in the absence of the ligand. The complete analyses of root mean square deviation (RMSD) are reported in Figures S3 and S4, revealing especially that the RMSD contributions are mainly borne by the terminals and the loop regions, which are very flexible.

**Figure 2:**
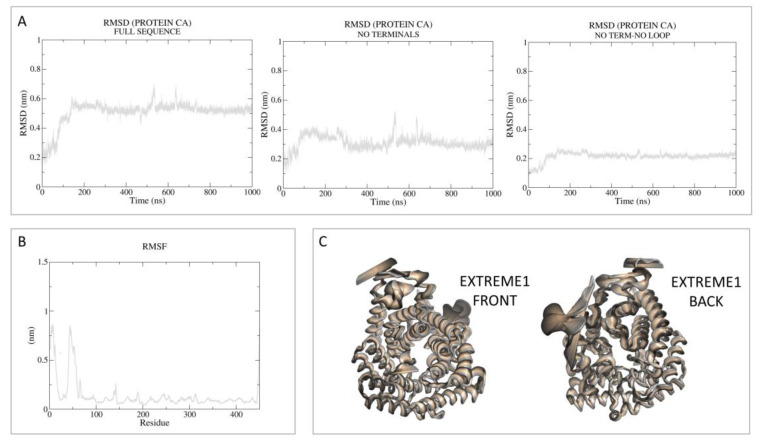
RMSD, RMSF, and PCA analyses performed on the apo-LANCL2 system (AF model; MODEL1) as LANCL2-replica 2. (**A**) RMSD of protein Cα, calculated on the full sequence, without terminals and without both terminals and the internal loop. (**B**) RMSF analysis per residue. (**C**) PCA was performed on protein Cα, to remove noise and visualize the most relevant movements of the target. PCA output is represented as the superposition of 50 states, expressing principal component 1 (extreme) as a color gradient (from dark to light).

**Figure 3:**
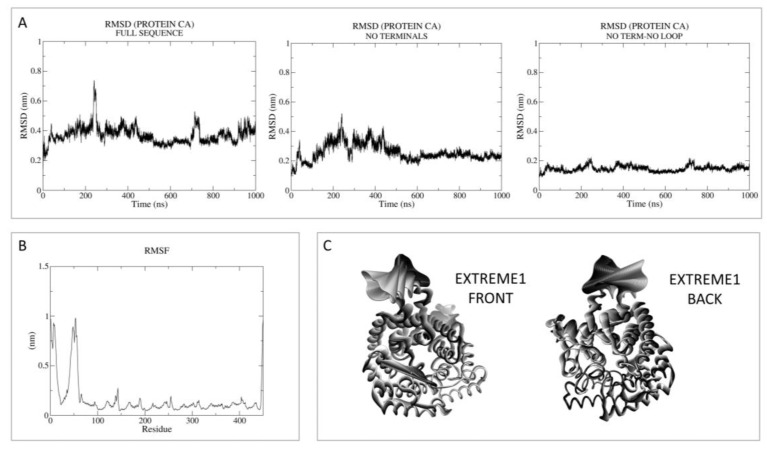
RMSD, RMSF, and PCA analyses performed on the apo-LANCL2 system (MOE model, MODEL2) as LANCL2-replica 2. (**A**) RMSD of protein Cα, calculated on the full sequence, without both terminals and without terminals and the internal loop. (**B**) RMSF analysis per residue. (**C**) PCA was performed on protein Cα, to remove noise and visualize the most relevant movements of the target. PCA output is represented as the superposition of 50 states, expressing principal component 1 (extreme) as a color gradient (from dark to light).

Then, principal component analysis (PCA) was performed to visualize the most important movement of the protein, removing “noise”, for the two models in Tables S8 and S9. In the present case, PCA was performed as a control with respect to the following holo-LANCL2 MD simulations. A schematic representation of these data, including the root mean square fluctuation (RMSF) values, is reported in Figure 2 and Figure 3.

As regards MODEL1 (AF), the dynamics quickly reach the RMSD plateau and it is possible to see that the mean value is lowered in the absence of the reconstructed terminals and loop (Figure 2). Based on the RMSF analysis, it is possible to notice that the most oscillating domains are the N-terminus and the in silico-built loop.

Concerning MODEL2 (MOE), the dynamics quickly reach the RMSD plateau, keeping the mean value very low in the absence of terminals and loop (Figure 3). Noisy peaks are also smoothed in this case, removing the most flexible regions. Accordingly, the RMSF analysis reveals the most oscillating areas as the N-terminus and the in silico-built loop.

From the reported analysis on the two sets of apo-simulations involving MODEL1 and MODEL2, no significant differences between the two models were highlighted and both of them were retained to perform the holo-simulations in the presence of **ABA**.

#### 3.1.2. LANCL2-**ABA** Molecular Docking and MD Simulations

**Figure 4:**
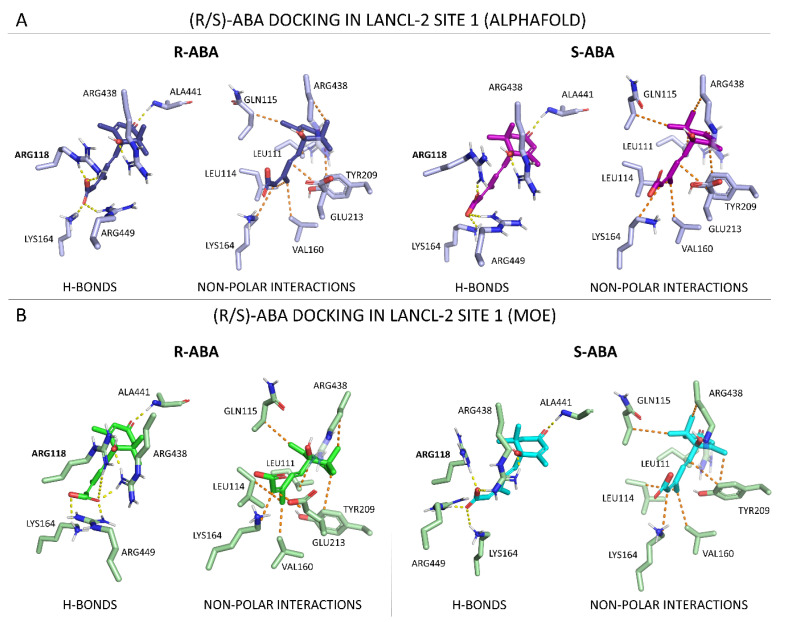
Docking poses of (*R/S*)-**ABA** in LANCL2. (*R/S*)-**ABA** in LANCL2 structure integrated with AlphaFold (MODEL1) (**A**). *R*-**ABA**: Blue, *S*-**ABA**: purple. (*R/S*)-**ABA** docked in LANCL2 with gaps completed with MOE (MODEL2) (**B**). *R*-**ABA**: green, *S*-**ABA**: cyan.

Based on the resulting molecular docking outcomes of *R/S*-**ABA** in LANCL2 Site 1, both *R*- and *S*-**ABA** shared a comparable positioning and pattern of interaction with the biological target, referring to both the two protein models (MODEL1–2). As shown in Figure 4, the two **ABA** enantiomers mainly displayed H-bonds with ARG118, LYS164, ALA441 (backbone), ARG438, and ARG449.

These complexes were submitted to the 1 μs MD simulations, and five replicas were produced for each of the four systems (*R-***ABA**:LANCL2, *S-***ABA**:LANCL2 via MODEL1, *R-***ABA**:LANCL2, *S-***ABA**:LANCL2 via MODEL2) to verify similarities/differences in the behavior of the two enantiomers. As shown in Figures S5 and S6, ligand RMSD analysis highlighted the overall stability of (*R/S*)-**ABA** in the high-affinity binding site (Site 1) at the LANCL2 MODEL1 and MODEL2, therefore being in agreement with the experimental data [30].

As a consequence, in the case of the MOE model (MOE; MODEL2), some replicas exhibit ligand destabilization (*R*-**ABA**:MODEL2 replica 4, *S*-**ABA**:MODEL2 replica 2, 4, and 5) and a single case of complete and definitive unbinding (*S*-**ABA**:MODEL2 replica 3). Using the AlphaFold model (AF; MODEL1), instead, only one replica shows a certain destabilization of the ligand (*S*-**ABA**:MODEL1 replica 1; see Figure S5) that was, however, able to find its way back to the binding site in a stable interaction with the protein.

Protein RMSD analyses of *R/S*-**ABA**:LANCL2 simulations involving MODEL1 (AF) and MODEL2 (MOE) are available as Figures S7–S10, respectively. The PCA analyses of the *R/S*-**ABA**:LANCL2 MD in MODEL1 (AF) and MODEL2 (MOE) are reported in Tables S10–S13. With respect to the previous PCA calculations involving the apo-LANCL2 MD simulations shown in Tables S8 and S9, this analysis did not reveal any evident discrepancies by comparing the PCA of apo- and holo-simulations in terms of the most important protein movement.

**Figure 5:**
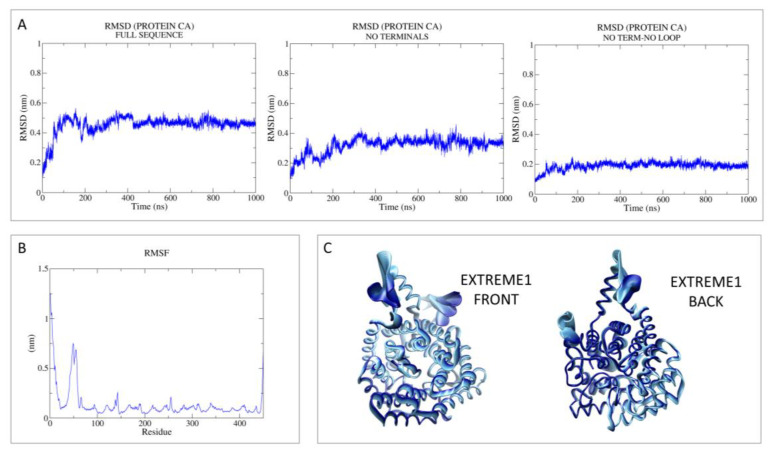
RMSD, RMSF, and PCA analyses performed on the R-**ABA**/LANCL2 system (AF model), as replica 1 case. (**A**) RMSD of protein Cα, calculated on the full sequence, without terminals and without terminals and the internal loop. (**B**) RMSF analysis per residue. (**C**) PCA was performed on protein Cα, to remove noise and visualize the most relevant movements of the target. PCA output is represented as the superposition of 50 states, expressing principal component 1 (extreme) as a color gradient (from dark to light).

**Figure 6:**
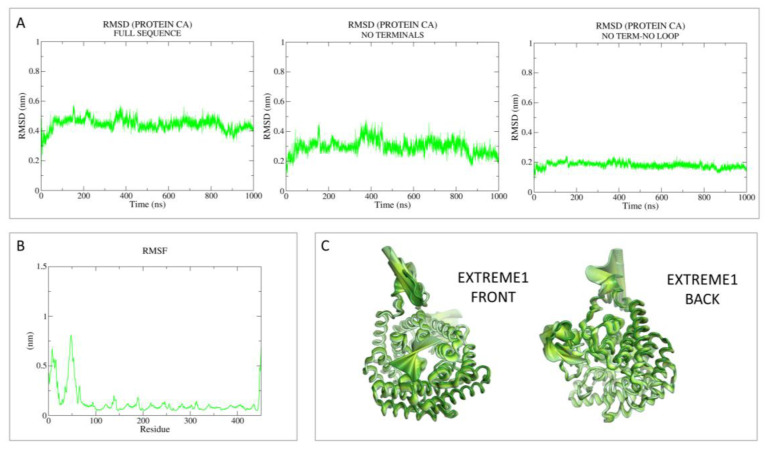
RMSD, RMSF, and PCA analyses performed on the R-**ABA**/LANCL2 system (MOE model), as replica 1 case. (**A**) RMSD of protein Cα, calculated on the full sequence, without terminals and without terminals and the internal loop. (**B**) RMSF analysis per residue. (**C**) PCA was performed on protein Cα, to remove noise and visualize the most relevant movements of the target. PCA output is represented as the superposition of 50 states, expressing principal component 1 (extreme) as a color gradient (from dark to light).

Figure 5 and Figure 6 give a perspective of the aforementioned analyses, focusing on the *R*-**ABA** enantiomer as the most stable. The dynamics quickly reach the RMSD plateau, and it is possible to see that the mean value is lowered in the absence of terminals and the loop.

The H-bond calculation and contact analyses were carried out between the ligand and the protein, and the results are reported in Figures S11–S14 for the *R/S*-**ABA**:LANCL2 (AF) and (MOE) simulations, respectively. A perspective of the most relevant H-bonds detected by the *R/S*-**ABA**:LANCL2 (AF) MD-based systems is reported in Tables S14 and S15.

According to this information, the *R*-**ABA**:LANCL2 system (AF model) featured stable H-bonds between **ABA** and ALA441, ARG438, and ARG118, and most of them were also retained in the *S*-**ABA**:LANCL2 system (AF-model). Indeed, the *S*-**ABA**:LANCL2 system (AF model) experienced a lower number of conserved interactions when compared to *R*-**ABA**. Only the H-bond with ALA441 and ARG118 were conserved in four/five replicas (replica 1 exhibited a certain destabilization). Other H-bonds, however, replaced the disrupted ones, and ligand binding is maintained in all of the cases. In particular, for replica 1, **ABA** cyclohexyl ring exhibits a kinked conformation, which disrupts the H-bond with ALA441. However, the **ABA** carbonyl establishes a very stable H-bond with TYR209 that still stabilizes the complex.

**Figure 7:**
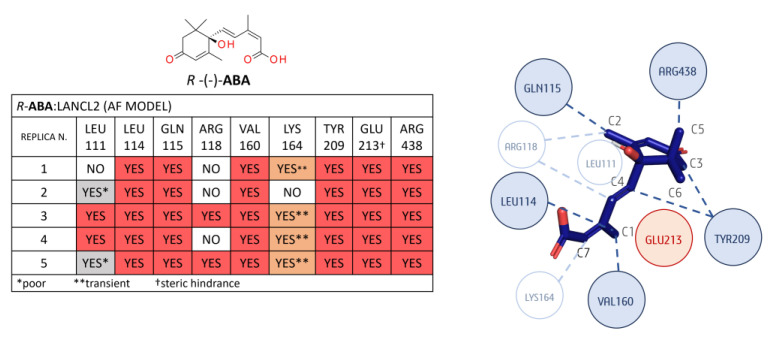
Non-polar contact analysis for the *R-***ABA**:LANCL2 (AF) complex, considering a radius of 3 Å from the ligand. In the associated table, the contacts highlighted in dark orange and light orange were found with an occupancy of at least 30%: the former are confirmed to be stable by visual inspection; conversely, the latter contacts in grey or light color exhibit a poor or transient character. A simplified diagram of the mentioned interactions is represented on the right side (replica 1). Dark blue circles represent the more conserved contacts throughout the five replicas, the light blue ones are less conserved, and the red circles represent steric hindrance role.

A detailed analysis of the *R/S*-**ABA**:LANCL2 system (AF model) in terms of non-polar binding events is reported as follows: Regarding the *R*-**ABA**:LANCL2 (AF) MD simulations, a non-polar interaction analysis was performed involving the ligand and the protein residues placed within 3 Å (Figure 7). No contacts below 30% of occupancy were considered.

The most relevant non-polar interactions involved residues LEU 114, GLN 115, VAL 160, TYR 209, and GLU 213, and ARG 438 hydrophobic moieties. More in detail, LEU 114 interacts with methyl C1 of **ABA**. The same **ABA** moiety also interacts with the VAL 160 side chain. GLN 115 aliphatic carbons interact with methyl C2 on the **ABA** cyclohexene. TYR 209 interacts with the **ABA** cyclohexene (C3) and the carbon before the double bond (C4).

In all replicas, a possible steric role of this residue is hypothesized, as the tyrosine plane is stacked before C3 of the cycle itself. Glu 213 laterally interacts with **ABA** via its Cβ and Cγ, but, more importantly, it seems to have an important steric role, as it is positioned before the geminal methyl groups (C5 and C6), preventing unbinding. Another conserved interaction is the London interaction between the C5 of **ABA** and the aliphatic chain of ARG 438. Other less conserved interactions involved LEU 111, ARG 118, and LYS 164. In replica 3 and 4, the LEU111 side chain interacts with the **ABA** cycle. This interaction is less stable in replicas 2 and 5. ARG118 Cδ (before the guanidine) is involved in non-polar contacts with the C2 of **ABA** and its double bond in replicas 3 and 5. A transient interaction is observed between the Cε of LYS164 and the **ABA** C7 in all the replicas except replica 2.

**Figure 8:**
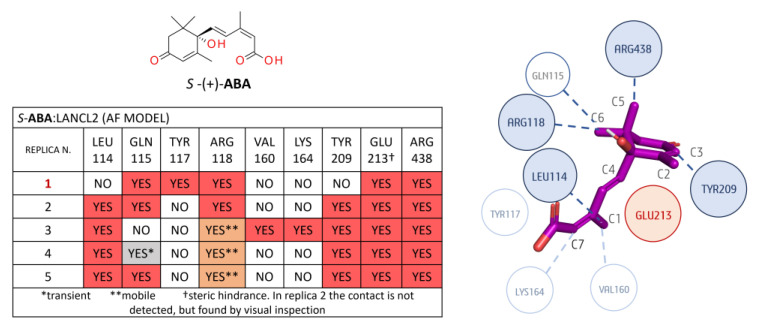
Non-polar contact analysis for the *S-***ABA**:LANCL2 (AF) complex, considering a radius of 3 Å from the ligand. More and less stable replicas are shown in black and red, respectively, in the associated table; contacts highlighted in dark orange and light orange were found with an occupancy of at least 30%: the former are confirmed by visual inspection as stable; conversely, the latter contacts, in grey and light color, exhibit a poor or transient character. A simplified diagram of the mentioned interactions is represented on the right side (replica 2). Dark blue circles represent the more conserved contacts throughout the five replicas, the light blue ones are less conserved, and the red circles represent steric hindrance role.

Concerning the non-polar contacts analysis of the *S*-**ABA**:LANCL2 system (AF model), only the interactions with residues ARG438, ARG118, and GLU213 are observed in the five replicas (see Figure 8), whereas, at variance with the *R*-**ABA**:LANCL2 simulations, the contacts with TYR117, VAL160, and LYS164 were almost completely lost.

Notably, ARG438 interacts with **ABA** C5, as in the case of the previously described *R*-**ABA**, while the **ABA** C6 interaction with ARG118 shows a certain variability among the replicas.

Steric hindrance of GLU213 is always detected, except in replica 2, as GLU213 was quite far from **ABA** (visual inspection). It is noteworthy that, in the first replica, a movement of this glutamic residue seems to be responsible for ligand destabilization, as it loses its steric role by assuming a different position, leaving more space for **ABA** movement. If we exclude replica 1, which exhibits a certain ligand instability, the interaction between LEU114 and **ABA** C1 is always present. Additionally, non-polar interactions with the TYR209 aromatic ring are observed in the same cases. The interaction of GLN115 with C6 is present in four out of five replicas, although it is transient in replica 4. In replica 1, an interaction is observed with TYR117, with this residue being in opposite positioning along the ligand unbinding. Interactions with VAL160 and LYS164 are relevant only in replica 3.

Then, the *R/S*-**ABA**:LANCL2 (MOE) MD-based systems were analyzed and the most relevant H-bonds contributing to **ABA**:LANCL2 binding were studied, giving similar results with respect to the AF model in the presence of *R*-**ABA** (Tables S16 and S17).

The most relevant H-bonds involving the *R*-**ABA**:LANCL2 system are established with ALA441, ARG438, and ARG118. Such interactions were replaced by H-bonds with TYR209 and ARG449 in replica 4, which exhibits ligand destabilization. H-bonds with LYS164 exhibits a transient character in all the simulated complexes.

As regards the *S*-**ABA** enantiomer, the only stable MD (replica 1) exhibits an interaction pattern coherent with the previous systems, involving H-bonds with ALA441, ARG118, and ARG438, and a transient H-bond with LYS164. The other replicas displayed only a single H-bond with TYR209, except replica 3, in which the simulation sampled an unbinding event.

**Figure 9:**
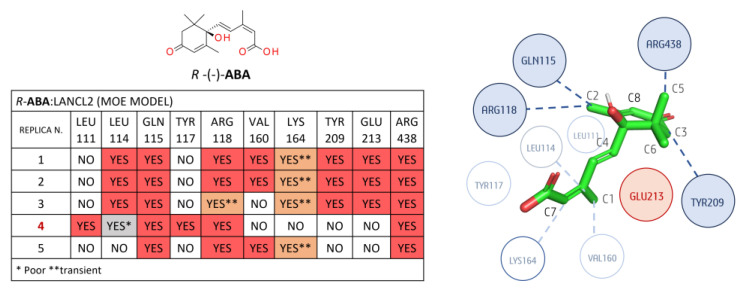
Non-polar contact analysis for the *R-***ABA**:LANCL2 (MOE) complex, calculated considering a radius of 3 Å from the ligand. More and less stable replicas are shown in black and red, respectively. In the associated table, contacts highlighted in dark orange and light orange were found with an occupancy of at least 30%; the former are confirmed as stable by visual inspection; conversely, the latter contacts in grey and light color exhibit a poor or transient character. A simplified diagram of the mentioned interactions is represented on the right side (replica 1). Dark blue circles represent the more conserved contacts throughout 5 the replicas, the light blue ones are less conserved, and the red circles represent steric hindrance role.

Concerning the *R*-**ABA**:LANCL2 non-polar interactions, residues GLN115, ARG118, TYR209, and ARG438 exhibit an interaction with **ABA** in all the replicas (see Figure 9). GLN115 interacts with **ABA** in the same manner as before in the AF model simulations (especially through Cβ). ARG 118 forms non-polar interactions between its Cδ and the C2 of **ABA**. TYR209 interacts as previously illustrated (the aromatic ring forms contacts with the inferior face of **ABA**). Again, ARG438 interacts through its aliphatic chain with C5 of **ABA** (Cβ and backbone).

Residue GLU213 displays a more external prominent positioning with respect to the AF model, and a moderately higher mobility. However, its steric hindrance is still present in replicas 1–3. In replica 4, this residue moves back toward the protein, and allows **ABA** to move, thus leading to ligand destabilization. In replica 5, the same residue moves towards the external part of LANCL2 while **ABA** rotates, altering the original binding pose. LEU114 methyl groups interact with **ABA** C1 in four replicas out of five. LYS164 shows once again the transient interaction between Cε and **ABA** C7 in four replicas out of five. In three replicas, a non-polar contact is observed between VAL160 and **ABA** C1. Replica 4 exhibits interactions involving LEU111 and the **ABA** cycle. This interaction is lost at the end on the trajectory, as **ABA** moves outwards.

**Figure 10:**
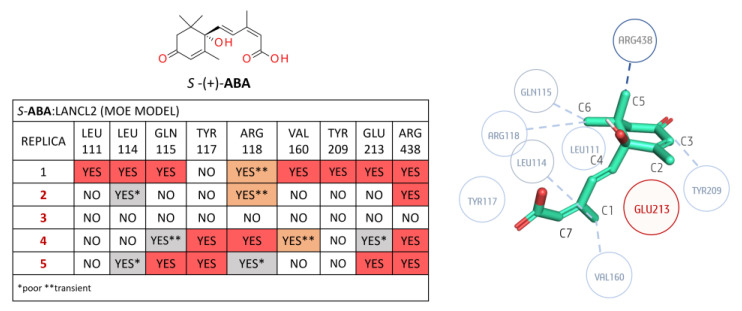
Non-polar contact analysis for the *S-***ABA**:LANCL2 (MOE) complex, considering a radius of 3 Å from the ligand. More and less stable replicas are shown in black and red, respectively. Dark orange and light orange contacts were found with an occupancy of at least 30%, being the first ones confirmed by visual inspection. Conversely, grey and light orange contacts exhibit a transient character. A simplified diagram of the mentioned interactions is represented on the right side (replica 1). Dark blue circles represent the more conserved contacts throughout the replicas, the light blue ones are less conserved, and the red circles represent steric hindrance role.

Analyzing the *S*-**ABA**:LANCL2 non-polar interactions, the only stable replica, i.e., #1, exhibits contacts with residues LEU111, LEU114, GLN115, ARG118, VAL160, TYR209, GLU213, and ARG438. The latter is present in all the simulations, with the exception of the third replica, in which an unbinding event occurs (see Figure 10).

Additionally, by a visual inspection, it is possible to see that this interaction seems to be important for unbinding prevention, as the aliphatic chain of ARG438 remains in contact with **ABA** methyl (interacting with the distal carbons of the side chain tethered to Cβ), although the ligand position is submitted to small destabilizations. This behavior suggests that interaction with this residue is relevant, if not even crucial/essential, for **ABA** binding, although not sufficient to keep the ligand strictly in place. In fact, in replicas 2, 4, and 5, **ABA** is able to display a compact conformation, exhibiting novel contacts within the binding site.

Interaction with GLN115 is observed in three replicas out of five, although replica four sampled a rotation of **ABA**, which is reflected in different areas of the ligand interacting with GLN115. Replicas 4 and 5 exhibit an interaction of **ABA** C1 with the TYR117 ring. The ARG118 aliphatic chain interacts with **ABA** C2 in replicas 1, 2, and 4, with a certain mobility. Replica 5 displays a parallel orientation of ARG118 with **ABA**, and the hydrophobic chain interacts with **ABA** double bond and C1. In addition to replica 1, VAL160 interacts with C1 in replica 4, but the interaction is destabilized with **ABA** rotation. The GLU213 steric role is maintained in replica 1. In replica 2, this residue moves laterally, but maintains its hindrance. In replica 3, a similar behavior is observed, but, in this case, the retreat of the residue and the rotation of the ligand allow **ABA** to bypass GLU213. A very similar situation also occurred in replica 4, although the simulation time was not sufficient for unbinding. In replica 5, the steric role of GLU213 is conserved along the whole simulation/trajectory.

As a result, the AF model gave rise to more stable binding with both *R*- and *S*-**ABA** with respect to the MOE model. It is possible to notice that, in all the stable dynamics, a key H-bond between the carbonyl of **ABA** cyclohexene and the backbone NH of ALA 441 is present (such as *R*-**ABA**:AF replica 1 shown in Figure S11). In destabilized dynamics (*S*-**ABA**:AF replica 1, *R*-**ABA**:MOE replica 4, and *S*-**ABA**:MOE replicas 2, 4, and 5), this H-bond is disrupted and the **ABA** carbonyl interacts with the TYR209 OH group (such as *R/S*-**ABA**:MOE in Figures S13 and S14). In these cases, complete unbinding is prevented. A similar behavior is kept by the H-bond established with residues ARG118, coherent with the previously reported mutagenesis experiments [30]. H-bonds by ARG438 were detected in the majority of the replicas. Sporadic H-bonds are observed with other residues, such as ARG449, LYS448, GLU213, and LYS164. An exception is represented by replica 3, in which a complete unbinding event occurred, and no H-bond occupancy was reached 20% of the time.

**Figure 11:**
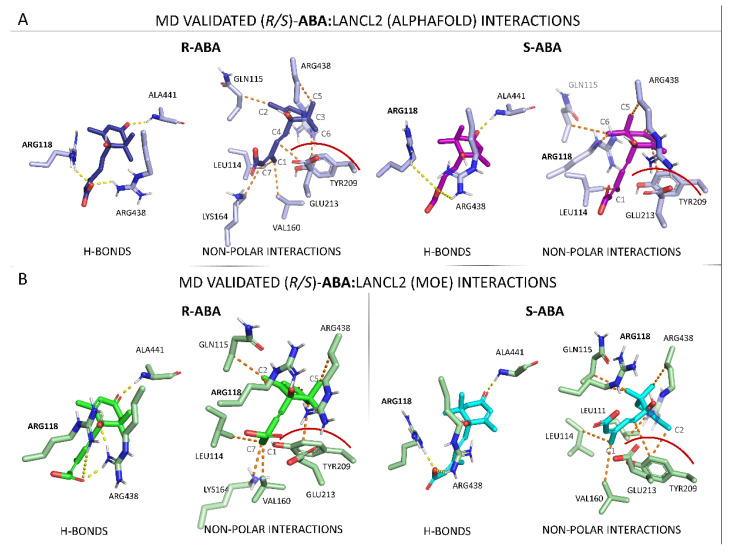
(*R/S*)-**ABA**:LANCL2 interactions (H-bonds and non-polar interactions) observed during the dynamics (replica 1). (**A**) *R/S*-**ABA** interactions with LANCL2 (AlphaFold model). (**B**) (*R/S*)-**ABA** interactions with LANCL2 (MOE model).

A visual perspective of the most important MD-validated interactions in both Model 1 and 2 are represented in Figure 11 (for clarity purposes, only the dynamics in which the ligand is stably bound were considered for the graphical representation).

Regarding the AlphaFold-completed LANCL2 structure (MODEL1), exhibiting greater stability than the MOE-completed one (MODEL2), several key contacts can be pointed out in order to explain this outcome: (i) a key salt bridge with ARG118, coherent with previously reported mutagenesis experiments [30], (ii) one H-bond involving ALA441 and the carbonyl moiety of **ABA** cyclohexyl substituent, (iii) several non-polar interactions with residues LEU114, GLN115, ARG118 (aliphatic chain), VAL160, TYR209, and ARG438, and (iv) common interactions shared by *R*-**ABA** and *S*-**ABA** involving the two geminal methyl groups and the aliphatic portion of ARG438, and the agonist main core TYR209, LEU114, and GLU213. By a visual analysis, it was seen that the GLU213 steric contribution is a key factor for **ABA** binding.

By visualizing the two models MODEL1–2 starting complexes, it is possible to notice that the IF procedure led to different side chain orientations in the two cases (MODELS 1–2), giving an explanation of the differences in the ligand-binding site positioning and stability when the two models are compared, as shown in Figure S15.

However, concerning the most stable binding positioning observed within the (*R/S*)-**ABA**:MOE replicas, the protein–ligand contacts are quite in accordance with the AlphaFold interaction analysis. The most important H-bonds (ARG118 and ALA441) are, in fact, also observed in this case. Additionally, a stabilizing non-polar interaction with ARG438 (aliphatic chain), TYR209 (aromatic ring), and GLN115 (C beta) are detected.

#### 3.1.3. **BT-11**:LANCL2 MD Simulations

Aiming at exploring the putative binding mode of the LANCL2 synthetic agonist **BT-11** [27] (whose pose remained yet undisclosed), further molecular docking and MD simulations have been performed. Thus, a docking run focused on LANCL2 Site 1 was performed and three docking poses were chosen for visual inspection (Figure S16A). Details of the obtained scoring functions are reported in Table S5.

Both pose 1 and 2 explore the same areas being H-bonded to the TYR209 and ARG118 (already mentioned several times), while pose 3 inserts the outer part of the ligand in a different groove on the right side of Site 1 (Figure S16B). To explore the most reliable **BT-11** poses, the three corresponding complexes were submitted to MD (two replicas of each pose, 1 μs long). According to the residence time of these initial simulations, **BT-11** pose 2 proved to be highly comparable to pose 1, both being more stable than pose 3 (Figure S16B). Indeed, pose 3 was immediately destabilized by many interaction losses and was, therefore, excluded from further analyses. On this basis, pose 1 was retained as the most promising pose for Site 1, and submitted to three further simulation replicas of the complex (for a total of five replicas, each 1 μs long) for its accurate characterization.

Regarding pose 1, the ligand RMSDs and ligand residence time are reported in Figure S17. It is possible to notice that a certain ligand stability is present, but that, in four out of five cases, binding is not preserved until the end of the runs. On the other hand, the protein-only RMSD of LANCL2 in complex with the **BT-11** docking pose 1 along the 1 μs-long MD simulation was quite stable (Figure S18). The corresponding complete analyses of **BT-11**:LANCL2 H-bonds and further (non-)polar contacts are reported in Figures S19 and S20.

**Figure 12:**
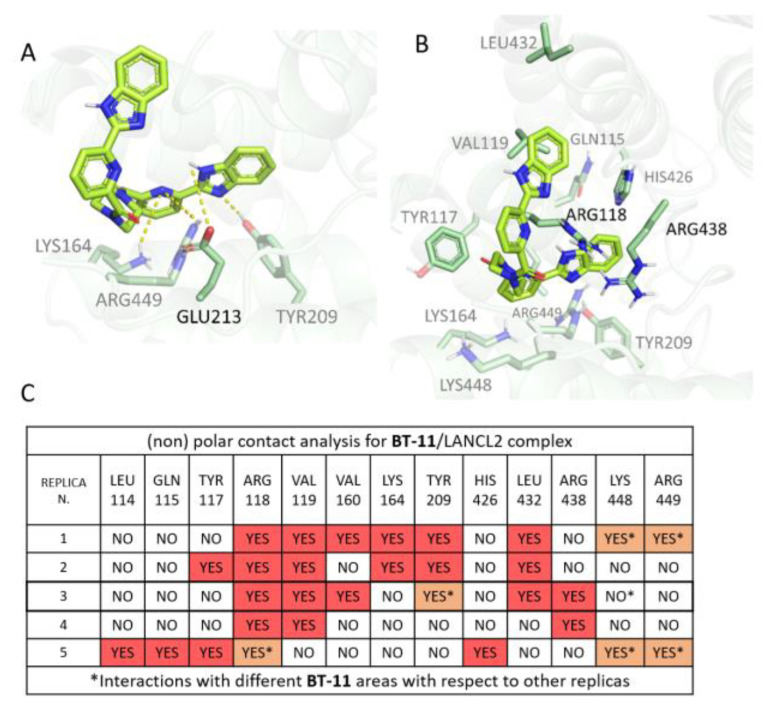
**BT-11**:LANCL2 recurrent H-bonds (**A**) and other (non-)polar contacts (**B**). The compound is reported in green and colored by atom types. Contact analyses table concerning (non-)polar contacts are also reported (**C**).

As shown in Figure S20, some H-bonds are detected, but none of them was conserved among the replicas. The only H-bond that exhibits a stable binding is the one involving GLU213 observed in replica 3 (Figure 12A). Such an H-bond is established with the NH on **BT-11** benzimidazole (internal area). Concerning other (non-)polar contacts featured by replica 3, it is possible to individuate conserved interactions between **BT-11** and VAL119, as well as with the hydrophobic side chain of ARG118, while the positive charge of this residue was engaged in cation–π contacts with the ligand benzimidazole ring (Figure 12B).

Our reference simulation (replica 3) also exhibits non-polar contacts with LEU432 and ARG438, as shared by replicas 1, 2, and 4, respectively (Figure 12C). LEU432 interacts with the external benzimidazole, stabilizing the outer part of the ligand. This interaction is also observed in replicas 1 and 2. ARG438 seems to establish a key contact, possibly a cation–π interaction. TYR209 is observed to interact with **BT-11** in replica 3, but, at variance with replicas 1 and 2, the ligand rotates and the two cyclic systems assume an orthogonal orientation. Replicas 2 and 5 exhibit a contact with the TYR117 ring, by the internal pyridine of **BT-11**. Two replicas (1 and 2) exhibit interaction with LYS164 Cε, again interacting through the internal **BT-11** pyridine ring. LYS448 and ARG449 were observed to interact with **BT-11** through several moieties, in replicas 1 and 5. The latter also exhibits interaction with LEU114, GLN115, and HIS426.

#### 3.1.4. Virtual Screening

The obtained MD results at an all-atom resolution provided a detailed picture that allowed us to evaluate in silico a novel putative LANCL2 ligand (**AR-42**), to be exploratorily investigated by biological assays. In accordance with the previous results, a putative LANCL2-ligand pharmacophore model should guarantee H-bonds with ARG118, ALA441, and a folded positioning to be properly surrounded by TYR205, TYR209, and GLU213.

**Figure 13:**
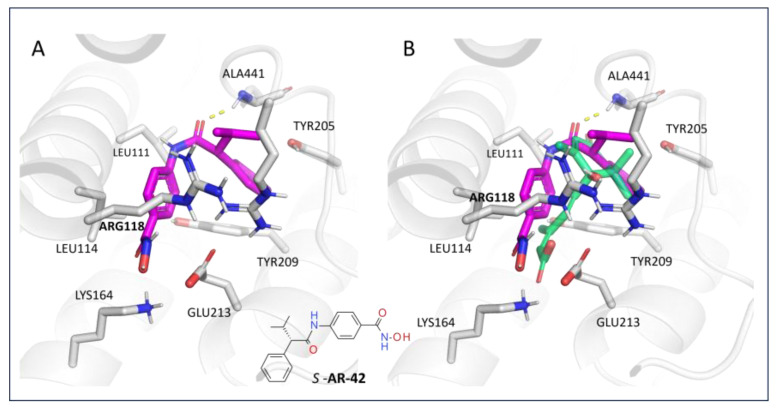
**AR-42** (C atom; magenta) binding pose alone within the LANCL2 Site 1 (**A**) and superimposed to **ABA** (C atom; light green) (**B**). The chemical structure of the ligand is reported.

Based on the aforementioned computational results, we evaluated in silico via pharmacophore-based VS a novel drug-like compound as a putative LANCL2-targeting derivative. We focused on the only compound **AR-42** among the known series of HDAC inhibitors based on (i) the presence of the hydroxamic moiety as a bioisostere of the **ABA** key carboxylic group tethered to (ii) a flat main core displaying comparable features, dimensions, and H-bonding groups with respect to **ABA** [67,68]. Our new hit compound (**AR-42**) was investigated via Maestro docking with the IF protocol (see Table S18 for scoring function results). The prevalent protonation state was reported. As shown in Figure 13A, the oxygen atom of the carboxamide group was engaged in H-bonds with ALA441, in a similar fashion to the reference compound **ABA**.

In addition, the branched substituent, including the alkyl group and the terminal phenyl ring, displayed promising van der Waals contacts and π−π stacking with ALA441, TYR205, and TYR209, respectively. This kind of positioning allowed the hydroxamic moiety and the tethered phenyl group to be well-oriented in the proximity of LEU114 and LYS164. As a result, **AR-42** featured a folded conformation endowed with comparable steric hindrance and electrostatic properties if compared to **ABA**. Indeed, the **AR-42** hydroxamic function and the opposite aromatic ring were superposed to the carboxylic and cyclohexyl portion of the reference compound (see Figure 13B).

### 3.2. Biochemical Assays

#### 3.2.1. Mitochondrial Function Analysis in LANCL2-Overexpressing and LANCL2-Silenced Cells

The computational studies have been accompanied by biochemical assays aimed at comparing the effect of **BT-11** and of the newly screened LANCL2 agonist (**AR-42**) with that of **ABA** on LANCL2-overexpressing (OVL2) or LANCL2-silenced (SHL2) H9c2 cardiomyocytes. LANCL2 overexpression and silencing were previously analyzed by Western blot experiments as described in [15] and resulted in a significant overexpression (45 times) and a 90% reduction of LANCL2 relative to control cells.

**Figure 14:**
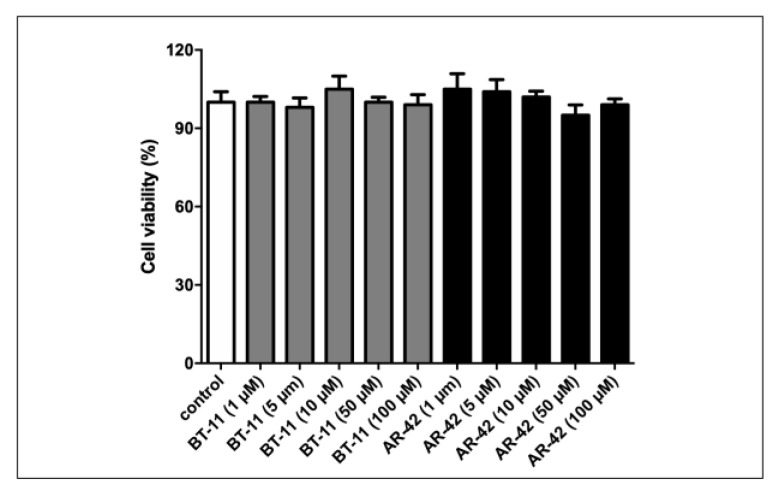
Cell vitality as determined by resazurin assay. H9c2 cells were incubated 24 h with 1, 5, 10, 50, and 100 µM **BT-11** or **AR-42**. Cell vitality was measured, adding resazurin (0.002% final concentration) and reading fluorescence at 590 nm. Results shown are the mean ± SD from three experiments.

At first, we tested the cell viability by a resazurin assay incubating H9c2 cells with **BT-11** or **AR-42** in parallel (1, 5, 10, 50, and 100 µM final concentration). Any significant reduction of viable cells was observed as compared with control cultures also at the highest concentrations tested (Figure 14). It is already known that **ABA** is non-cytotoxic and has already been used for in vivo experiments even at high concentrations [9].

LANCL2-overexpressing (OVL2) or LANCL2-silenced (SHL2) H9c2 cardiomyocytes cells were then incubated in parallel with **ABA**, **BT-11**, and **AR-42** (10 µM for 4 h), and mitochondrial function analysis and gene expression quantification were performed as follows.

**Figure 15:**
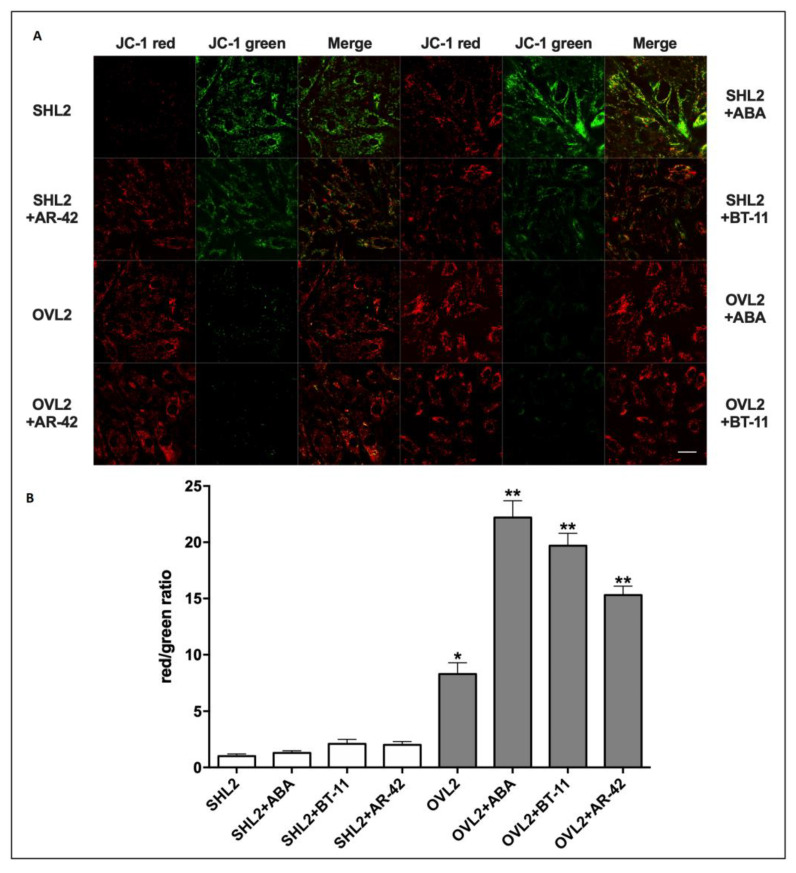
**ABA**, **BT-11**, and **AR-42** increased mitochondrial proton gradient in LANCL2-overexpressing vs. LANCL2-silenced H9c2. H9c2 cardiomyocytes overexpressing LANCL2 (OVL2) or LANCL2-silenced (SHL2) cardiomyocytes were loaded with the mitochondrial proton gradient (ΔΨ)-sensitive fluorescent dye JC-1, which changes its emission from green to red with an increase of ∆Ψ (λex 488 nm, λem 527 nm for green fluorescence, and at 590 nm for red fluorescence). (**A**) Representative confocal microscopy images of the cells. Merged images show JC-1 both as monomer (green) and as aggregates (red). The effect of **ABA**, **BT-11**, and **AR-42** on the mitochondrial ΔΨ was explored in LANCL2-overexpressing vs. LANCL2-silenced cells. (**B**) Red/green fluorescence ratio calculated for each experiment shown in panel A. The mean ± SD of the red/green fluorescence ratio was always calculated in at least three microscopic fields (scale bar: 20µm). * *p* < 0.05 and ** *p* < 0.01 relative to the respective SHL2 cells by unpaired *t*-test.

The mitochondrial function on LANCL2-overexpressing and LANCL2-silenced cardiomyocyte cells treated with the **ABA**, **BT-11**, and **AR-42** molecule was investigated using the mitochondrial proton gradient (∆Ψ)-sensitive dye JC-1. This fluorescent molecule accumulates within mitochondria and changes its emission from green to red as the ∆Ψ increases [15,32]. Mitochondrial fluorescence was largely green, with just a trace of red, in the LANCL2-silenced cells; conversely, mitochondrial fluorescence was predominantly red in the LANCL2-overexpressing cells; the calculated red/green ratio was almost 1 log higher in the overexpressing vs. silenced cells, as already described before [15]. Next, the effect of **ABA**, **BT-11**, and **AR-42** on the mitochondrial ∆Ψ was explored in LANCL2-overexpressing vs. LANCL2-silenced cells: we observed a higher red/green ratio in **ABA**-, **BT-11**-, and **AR-42**-treated overexpressing vs. silenced cells; conversely, the addition of **ABA**, **BT-11**, and **AR-42** (10 μM final concentration) only slightly increased the red fluorescence in silenced cells; in LANCL2-overexpressing cells treated with **ABA**, **BT-11**, and **AR-42**, the red fluorescence was more visible and the calculated red/green ratio had increased by approximately 2.5-fold compared with LANCL2-overexpressing not-treated cells (see Figure 15).

The higher mitochondrial ∆Ψ observed in **ABA**-, **BT-11**-, and **AR-42**-treated, LANCL2-overexpressing cells indicates an improved mitochondrial performance and suggests that the activation of LANCL2 by specific agonists could be important for the maintenance of the mitochondrial function and, consequently, for cardiomyocyte energy production. Indeed, LANCL1/2-overexpression in cardiomyocytes improves several structural and metabolic features of the cells, including contractile protein expression, ATP synthesis and oxidative metabolism, and cell cycle regulation [16].

#### 3.2.2. qPCR Analysis

**Figure 16:**
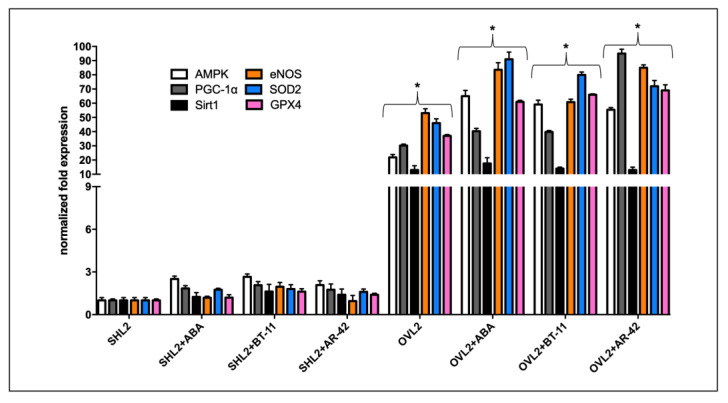
**ABA**, **BT-11**, and **AR-42** increased transcription of genes related to mitochondrial function in LANCL2-overexpressing H9c2. The gene transcripts analyzed by qPCR were the AMPK/PGC-1α/Sirt1 axis, which controls mitochondrial function, and eNOS that produces cardioprotective NO and SOD2 and GPX4, which protect from oxidative stress. Overexpressing LANCL2 (OVL2) or LANCL2-silenced cells (SHL2) were incubated in the absence or in the presence of 10 µM **ABA**, **BT-11**, or **AR-42** for 4 h. After incubation, total RNA was extracted and cDNA was synthesized starting from 1 µg of total RNA and was used as a template for qPCR analysis. Results are expressed relative to untreated silenced LANCL2 cardiomyocytes. * *p* < 0.01 relative to the respective SHL2 cells by unpaired *t*-test.

We analyzed the gene transcription levels of AMPK, PGC-1α, and Sirt1 (regulators of mitochondrial biogenesis and function), of eNOS (responsible for the production of cardioprotective NO), and of two ROS-scavenging enzymes, superoxide dismutase (SOD2) and glutathione peroxidase (GPX4) on LANCL2-overexpressing and LANCL2-silenced H9c2 cardiomyocytes treated or not with **ABA**, **BT-11**, and **AR-42**. The results show that LANCL2 overexpression highly stimulated AMPK, PGC-1α, Sirt1, and eNOS, as already observed before [15]; in addition, we observed a higher transcription of SOD2 and GPX genes in overexpressing compared to silenced cells, and treatment with **ABA**, **BT-11**, or **AR-42** further increased the mRNA levels of these genes (Figure 16).

Recently, the **ABA**/LANCL system has been shown to control fundamental mechanisms in the response of cardiomyocytes to hypoxia/reoxygenation, through the activation of the AMPK/PGC-1α/Sirt1 axis, by increasing NO generation and eNOS transcription, expression, and phosphorylation; and stimulating mitochondrial respiration, the fatty acid-fueled respiration rate, and the transcription and expression of contractile and ion channel proteins. [15,16]; in addition, LANCL1/2-overexpressing cells have a significantly reduced mitochondrial ROS content, higher expression levels of ROS-scavenging enzymes, and decreased levels of radical-generating enzymes compared with double-silenced cells (Sturla L. et al., manuscript under revision), suggesting a transcriptional control by the **ABA**/LANCL system on cardiomyocyte mechanisms of resistance to oxidative stress. **BT-11**, as well as the newly screened LANCL2 agonist (**AR-42**), show a similar stimulatory effect on the transcription of all genes analyzed in LANCL2-overexpressing cardiomyocytes.

## 4. Discussion

Recently, the X-ray structure of apo-LANCL2 (6WQ1) was published. Here, we exploited this experimental structural information to elucidate the binding pose of (*R/S*)-**ABA** in its high-affinity binding site. These data were used to guide the design of novel LANCL2 activators. In more detail, we modelled the gaps of the crystal structure of LANCL2 with AlphaFold and MOE. The two alternatives were used to perform the docking of *R/S*-**ABA** in Site 1 (see previous Figure 5). In both the models, the enantiomers displayed similar modes of interaction with the target. These complexes were tested along five replicas of 1 μs-long MD simulation for each of the four systems (*R-***ABA**:LANCL2 (AlphaFold), *S-***ABA**:LANCL2 (AlphaFold), *R-***ABA**:LANCL2 (MOE), and *S-***ABA**:LANCL2 (MOE)). The resulting trajectories were analyzed to clarify the protein–ligand interaction pattern. Noticeably, the most important interactions characterizing *R-* and *S-***ABA**/LANCL2 binding are the same for the two enantiomers. In particular, the most important interactions were the key H-bonds with residues ALA441 and ARG118, and an important steric hindrance between GLU213 and the methyl-substituents of **ABA** cyclohexene. Indeed, the presence of these methyl groups gives very similar interactions, as the geminal equatorial methyl is able to mimic the **ABA** single equatorial methyl and vice versa. Such a methyl points toward a region (the side chain of ARG438) which can be reached from both **ABA** enantiomers. These results explain, from a chemical perspective, why both enantiomers are functionally active in mammalian cells [13], a perplexing fact regarding the specificity of the **ABA**-LANCL interaction. Now, we know that, indeed, most chemical interactions stabilizing the two **ABA** enantiomers in their binding site are similar.

Moreover, *R-***ABA** interacts with LEU114 and GLN115. In the majority of the cases, interactions with VAL160 are observed. TYR 209 and GLU 213 appear to have a steric hindrance role, which, in the case of GLU 213, seems to be key for **ABA** stabilization. Another important contact is observed with the aliphatic chain of ARG 438.

*S-***ABA** exhibits interaction with ARG438, LEU114, and GLN115, as well as with ARG 118. In this case, again, steric hindrance from GLU213 seems to be key for binding. In particular, the steric factor plays a crucial role in the stability differences observed for the two enantiomers, as *R-***ABA** is more hindered in the right part of the binding site with respect to the *S*- enantiomer, being much more capable of moving and rotating from the stable binding pose. The change in the complex stability by passing from the AF to MOE completed structures seems to be related to the different position of the C-terminal loop (which was previously modelled in silico).

From a drug design perspective, it seems that one or more methyl groups on the **ABA** cyclohexene properly oriented towards the protein crevice is an essential requirement for affinity. In the case of the *S-***ABA** enantiomer, a single methyl was efficiently placed within the LANCL2 cavity, leading to the agonist’s higher instability with respect to *R-***ABA**.

In addition to **ABA**, another known LANCL2 activator was considered (**BT-11**). In particular, in the absence of an experimentally validated, publicly available complex structure, we speculated that this synthetic activator may bind to the same binding site as **ABA**, as the exerted function is the same (LANCL2 activation). We, therefore, applied a three-step (SP, XP, and IF) docking protocol to **BT-11** and selected three poses for MD validation. In light of the result obtained thus far, more studies are required to better clarify the **BT-11**:LANCL2 interaction mode, as Site 1 simulations lead to non-obvious scenarios. In particular, it is possible that Site 1 is not exactly the precise correct site for **BT-11**. Another explanation is that static parametrization and the parallel ligand–protein preparation do not allow us to properly consider the possible induced interactions that may be formed between the ligand and the protein; for example, the cation–π interaction between the benzimidazole moiety of **BT-11** and the surrounding arginine residues. Moreover, an important factor is represented by the solvent exposed part of the ligand, whose movements may lead to a destabilization. It is possible that the chosen poses do not carry sufficient stabilization of this part, which may be reached by performing a more in-depth docking study. It is important to note that correlations between residence time in MD simulations and affinity measures are not always easy to observe, even due to the forces not explicitly included in the FF (e.g., cation–π interaction, or π−π stacking). Additionally, the necessary time for a ligand to produce its biological effect is often unknown. On this basis, we can only affirm that further investigation is needed to clarify the **BT-11** binding mode.

Nevertheless, these computational studies allowed us to define a pharmacophore, involving two H-bonds (ARG118 and ALA441) and a steric hindrance behind GLU213, whose use in a screening campaign allowed us to individuate compound **AR-42**. To preliminarily support and validate the aforementioned computational studies, the effect of **ABA** and **BT-11**, as well as of the newly screened LANCL2 agonist **AR-42**, has been evaluated on the mitochondrial proton gradient and on the transcription of specific genes, previously identified as targets of the **ABA**/LANCL hormone/receptor system in H9c2 cardiomyocytes [15,16]. The results obtained suggest that both **BT-11** and **AR-42**, as already observed for **ABA**, improved via LANCL2 the mitochondrial proton gradient (ΔΨ) and increased the transcription of the AMPK/PGC-1α/Sirt1 axis known to control energy metabolism, mitochondrial respiration, and biogenesis in the skeletal muscle [69] and heart [16,70]. Moreover, both **BT-11** and **AR-42** increased the expression of NO-generating eNOS and of ROS-scavenging enzymes. These results, which certainly need further in-depth experimental investigation both in vitro and in vivo, suggest that LANCL2 agonists could represent a new promising pharmacologic approach for improving mitochondrial function and counteract ROS-mediated inflammation and oxidative stress, which is involved in several disease conditions, such as metabolic disorders, cardiovascular and respiratory diseases, neurodegenerative disorders, and autoimmune and auto-inflammatory diseases [71]. In particular, protein expression of the gene transcripts and ROS levels in **BT-11**- and **AR-42**-treated cells need to be investigated together with mitochondrial respiration and oxidative metabolism. Nonetheless, the observations that these LANCL2-agonists activate the transcription of the AMPK/PGC-1α/Sirt1 axis (the master regulator of mitochondrial function) and increase the mitochondrial proton gradient (arguably the ultimate indicator of improved mitochondrial function) promise future interesting results in the field of cardioprotection.

## 5. Conclusions

Up until now, limited information has been available regarding the LANCL2 agonist binding event and mechanism of action, representing a hurdle in the design of novel effective ligands. Herein, we performed an in silico study involving docking experiments and MD simulations, exploiting the recently published X-ray structure of the target. In particular, the binding mode of *R/S*-**ABA** in its previously validated high-affinity binding site was studied, looking for key drug–target interactions and ligand-induced dynamic changes. Similarly, the LANCL2 agonist **BT-11** has been explored to highlight the main protein–ligand interactions to be fulfilled in the search for novel agonists. The results allowed us to test in silico **AR-42** as a new putative LANCL2 ligand, based on the compound features and docking mode. Biological assays seem to support the computational results, suggesting that, similarly to **ABA**, **BT-11** and **AR-42** could improve via LANCL2 the mitochondrial proton gradient of rat H9c2 cardiomyocytes and increase the transcription of the AMPK/PGC-1α/Sirt1 axis controlling the mitochondrial function in cardiac and skeletal muscle cells. 

## Data Availability

The data are contained within the article.

## Supplementary Materials

The following supporting information can be downloaded at: https://www.mdpi.com/article/10.3390/pharmaceutics15122754/s1, Figure S1: Sequence alignment of the human LANCL1/2 proteins; Figure S2: Complete structures of LANCL2. (A) Gaps filled with AlphaFold, front and lateral view. (B) Gaps filled with MOE, front and lateral view; Figure S3: Protein RMSD of AlphaFold-completed LANCL2 (apo-form) along the 1 μs MD simulation; Figure S4: Protein RMSD of the MOE-completed LANCL2 (apo-form) along the 1 μs MD simulation; Figure S5: Ligand RMSD for (*R/S*)-**ABA**:LANCL2 (AF; MODEL1) complex; Figure S6: Ligand RMSD for (*R/S*)-**ABA**:LANCL2 complex (MOE model, MODEL2). *R*-**ABA**: green, *S*-**ABA**: cyan; Figure S7: Protein RMSD of the AF-completed LANCL2 in complex with *R-***ABA** along the 1 μs MD simulation; Figure S8: Protein RMSD of the AF-completed LANCL2 in complex with *S-***ABA** along the 1 μs MD simulation; Figure S9: Protein RMSD of the MOE-completed LANCL2 in complex with *R-***ABA** along the 1 μs MD simulation; Figure S10: Protein RMSD of the MOE-completed LANCL2 in complex with *S-***ABA** along the 1 μs MD simulation; Figure S11: H-bond contacts analysis of the *R-***ABA**:LANCL2 (AF) MD simulations; Figure S12: H-bond contacts analysis of the *S-***ABA**:LANCL2 (AF) MD simulations; Figure S13: H-bond contacts analysis of the *R-***ABA**:LANCL2 (MOE) MD simulations; Figure S14: H-bond contacts analysis of the *S-***ABA**:LANCL2 (MOE) MD simulations; Figure S15: Comparison between the starting complexes of (*R/S*)-**ABA** in the two models (AF and MOE); Figure S16: Three **BT-11** docking poses were selected via molecular docking at the LANCL2 site 1; Figure S17: Ligand RMSD and residence time for **BT-11**/LANCL2 complexes; Figure S18: Protein RMSD of LANCL2 in complex with **BT-11** (docking pose 1) along the 1 μs MD simulation; Figure S19: H-bond contacts analysis of the **BT-11**:LANCL2 (AF) MD simulations; Figure S20: **BT-11** H-bond analysis table and representation. Table S1: *R-***ABA** docking scores as obtained via Alphafold (AF)-completed model; Table S2: *S-***ABA** docking scores as obtained via Alphafold (AF)-completed model; Table S3: *R-***ABA** docking scores as obtained via MOE-completed model; Table S4: *S-***ABA** docking scores as obtained via MOE-completed model; Table S5: **BT-11** docking scores as obtained via AF-completed model; Table S6: Description of the three performed MD equilibrations; Table S7: Primer sequences used for qPCR analyses. TableS8: Apo-LANCL2 (AlphaFold model; MODEL1) PCA analysis; Table S9: Apo-LANCL2 (MOE model; MODEL2) PCA analysis; Table S10: *R-***ABA**:LANCL2 (AlphaFold model) PCA analysis; Table S11: *S-***ABA**:LANCL2 (AlphaFold model) PCA analysis; Table S12: *R-***ABA**:LANCL2 (MOE model) PCA analysis; Table S13: *S-***ABA**:LANCL2 (MOE model) PCA analysis; Table S14: H-bond analysis for the *R*-**ABA**:LANCL2 (AF) complex. The corresponding occupancy values are shown as percentage; Table S15: H-bonds analysis for the *S*-**ABA**:LANCL2 (AF) complex. The corresponding occupancy values are shown as percentage; Table S16: H-bonds analysis for the *R*-**ABA**:LANCL2 (MOE) complex. The corresponding occupancy values are shown as percentage; Table S17: H-bonds analysis for the *S*-**ABA**:LANCL2 (MOE) complex. The corresponding occupancy values are shown as percentage; Table S18: **AR-42** docking scores as obtained via Alphafold (AF)-completed model.
