# Supplementary material for: New Insights into the LANCL2-ABA Binding Mode towards the Evaluation of New LANCL Agonists
Source: Pharmaceutics. 2023 Dec 12;15(12):2754. doi: 10.3390/pharmaceutics15122754 (PMC10747503; doi:10.3390/pharmaceutics15122754)
Supplement: Supplementary file 1 [file pharmaceutics-15-02754-s001.zip › pharmaceutics-2714733-supplementary.pdf]

SUPPORTING INFORMATION

New insights into the LANCL2-ABA binding mode towards the evaluation of new LANCL-agonists.

Naomi Scarano[1], Francesco di Palma[2], Nicola Origlia[3], Francesca Musumeci[1], Silvia Schenone[1], Sonia Spinelli[4], Mario Passalacqua[5], Elena Zocchi[5], Laura Sturla[5*], Elena Cichero[1*&] and Andrea Cavalli[2, &]

[1] Department of Pharmacy, Section of Medicinal Chemistry, School of Medical and Pharmaceutical Sciences, University of Genova, Viale Benedetto XV, 3, 16132 Genoa, Italy; naomi.scarano@edu.unige.it (N.S.); francesca.musumeci@unige.it (F.M.); silvia.schenone@unige.it (S.Sc.)

[2] Computational & Chemical Biology, Fondazione Istituto Italiano di Tecnologia, Via Morego 30, I-16163 Genova, Italy; francesco.DiPalma@iit.it (F.D), andrea.cavalli@iit.it (A.C.)

[3] National Research Council (CNR) Institute of Neuroscience, Pisa 56124, Italy; Nicola.origlia@in.cnr.it (N.O.)

[4] IRCCS Istituto Giannina Gaslini, Laboratorio di Nefrologia Molecolare, Via Gerolamo Gaslini 5, 16147 Genova, Italy soniaspinelli@gaslini.org (S.Sp.)

[5] Department of Experimental Medicine, Section of Biochemistry, University of Genoa, Viale Benedetto XV 1, 16132 Genova, Italy; ezocchi@unige.it (E.Z.); mario.passalacqua@unige.it (M.P.)

[6] Department of Pharmacy and Biotechnology, University of Bologna, Via Belmeloro 6, I-40126 Bologna, Italy

* Correspondence: elena.cichero@unige.it (E.C.); Tel.: +39-010-353-8350 (E.C.); laurasturla@unige.it (L.S.); Tel.:+39-010-353-8131

& Equally contributing Senior Authors

```
Query: 6WQ1_1|Chains A, B, C, D|LanC-like protein 2|Homo sapiens (9606) Query ID:
lcl|Query_55229 Length: 450

Sbjct>8D19_1|Chains A, B|Glutathione S-transferase LANCL1|Homo sapiens (9606)
Sequence ID: Query_55231 Length: 419

Query  17   AEMEERAFVNPFPDYEAAAGALLASGAAEETGCVRPPATTDEPGLPFHQDGKIIHNFIRR  76
            + M +RAF NP+ DY  +    LA G                    F   G++   F +R
Sbjct  19   SHMAQRAFPNPYADYNKS----LAEGY-------------------FDAAGRLTPEFSQR  55

Query  77   IQTKIKDLLQQMEEGLKTADPHDCSAYTGWTGIALLYLQLYRVTCDQTYLLRSLDYVKRT  136
            +  KI++LLQQME GLK+ADP D + YTGW GIA+LYL LY V  D  YL  +  YVK++
Sbjct  56   LTNKIRELLQQMERGLKSADPRDGTGYTGWAGIAVLYLHLYDVFGDPAYLQLAHGYVKQS  115

Query  137  LRNLNGRRVTFLCGDAGPLAVGAVIYHKLRSDCESQECVTKLLQLQRSVVCQESDLPDEL  196
            L  L  R +TFLCGDAGPLAV AV+YHK+ ++ ++++C+T+L+ L +     +   P+E+
Sbjct  116  LNCLTKRSITFLCGDAGPLAVAAVLYHKMNNEKQAEDCITRLIHLNKI----DPHAPNEM  171

Query  197  LYGRAGYLYALLYLNTEIGPGTVCESAIKEVVNAIIESGKTLSREERKTERCPLLYQWHR  256
            LYGR GY+YALL++N   G   + +S I+++   I+ SG+ L+R+   T + PL+Y+W++
Sbjct  172  LYGRIGYIYALLFVNKNFGVEKIPQSHIQQICETILTSGENLARKRNFTAKSPLMYEWYQ  231

Query  257  KQYVGAAHGMAGIYYMLMQPAAKVDQETLTEMVKPSIDYVRHKKFRSGNYPSSLSNETDR  316
            + YVGAAHG+AGIYY LMQP+ +V Q  L  +VKPS+DYV   KF SGNYP  + +  D
Sbjct  232  EYYVGAAHGLAGIYYYLMQPSLQVSQGKLHSLVKPSVDYVCQLKFPSGNYPPCIGDNRDL  291

Query  317  LVHWCHGAPGVIHMLMQAYKVFKEEKYLKEAMECSDVIWQRGLLRKGYGICHGTAGNGYS  376
            LVHWCHGAPGVI+ML+QAYKVF+EEKYL +A +C+DVIWQ GLL+KGYG+CHG+AGN Y+
Sbjct  292  LVHWCHGAPGVIYMLIQAYKVFREEKYLCDAYQCADVIWQYGLLKKGYGLCHGSAGNAYA  351

Query  377  FLSLYRLTQDKKYLYRACKFAEWCLDYGAHGCRIPDRPYSLFEGMAGAIHFLSDVLGPET  436
            FL+LY LTQD KYLYRACKFAEWCL+YG HGCR PD P+SLFEGMAG I+FL+D+L P
Sbjct  352  FLTLYNLTQDMKYLYRACKFAEWCLEYGEHGCRTPDTPFSLFEGMAGTIYFLADLLVPTK  411

Query  437  SRFPAFEL  444
            +RFPAFEL
Sbjct  412  ARFPAFEL  419
```

Figure S1. Sequence alignment of the human LANCL1/2 proteins via BLAST Blosum62 matrix (Altschul, S F et al. Basic local alignment search tool. Journal of molecular biology vol. 215,3 (1990): 403-10. doi:10.1016/S0022-2836(05)80360-2). Missing gap in LANCL2 is highlighted in red.

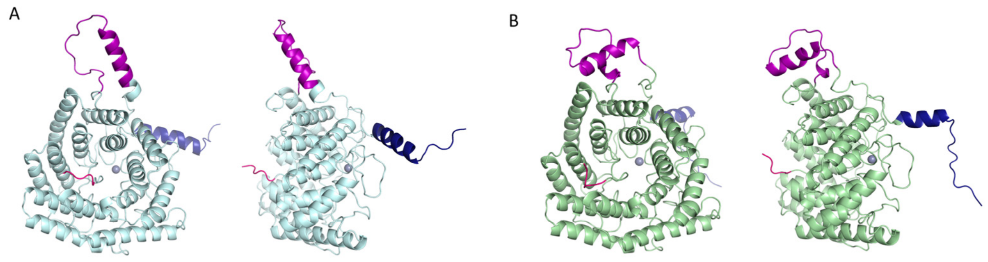

Figure S2. Complete structures of LANCL2. (A) Gaps filled with AlphaFold, front and lateral view. (B) Gaps filled with MOE, front and lateral view. Blue: N-terminus, Hotpink: C-terminus, Purple: modelled loop.

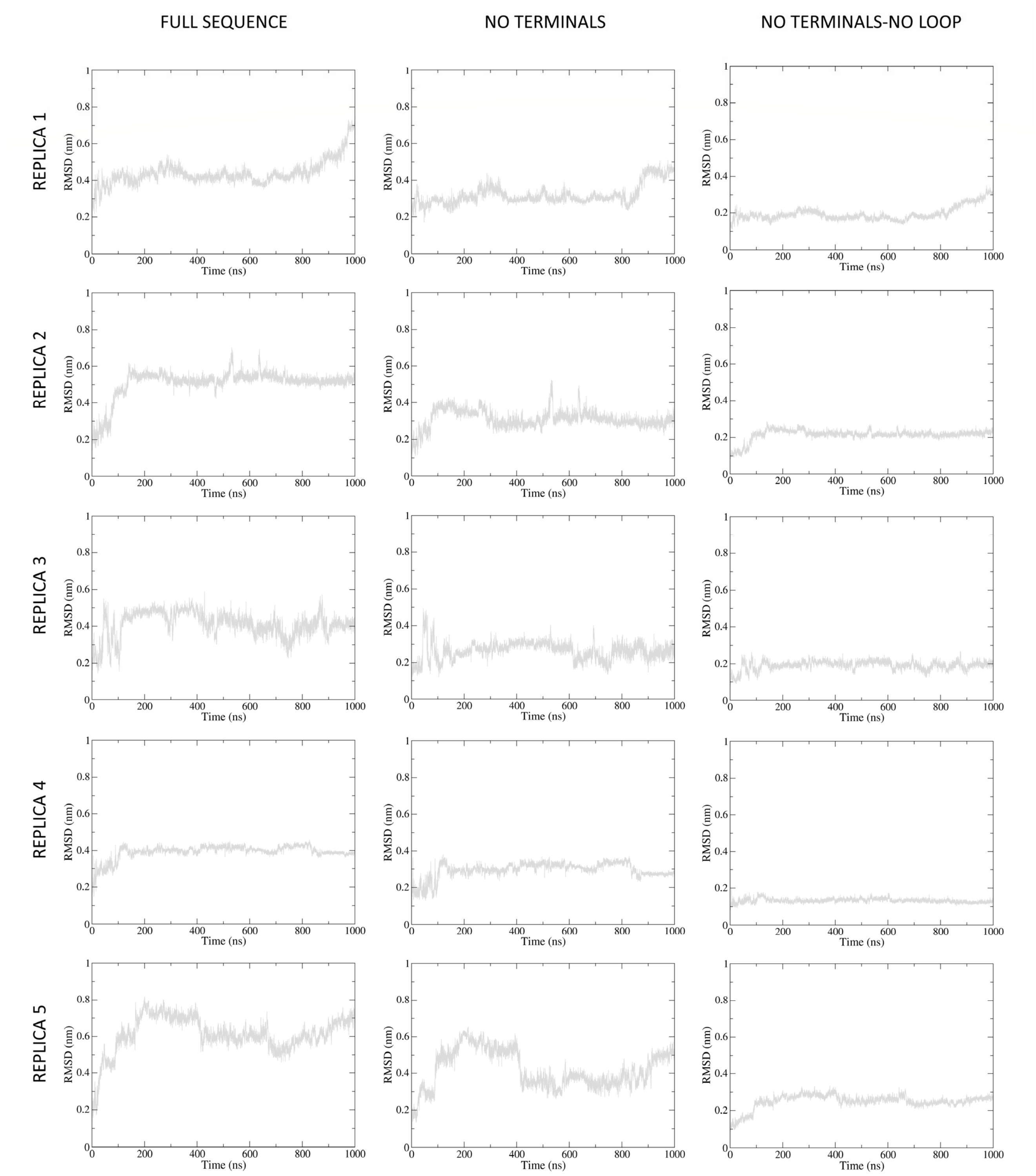

Figure S3. Protein RMSD of AlphaFold-completed LANCL2 (apo-form) along the 1 μs MD simulation. The RMSD was calculated on protein Cα and for the full sequence, removing the terminals, and removing terminals and the internal loop (34-60).

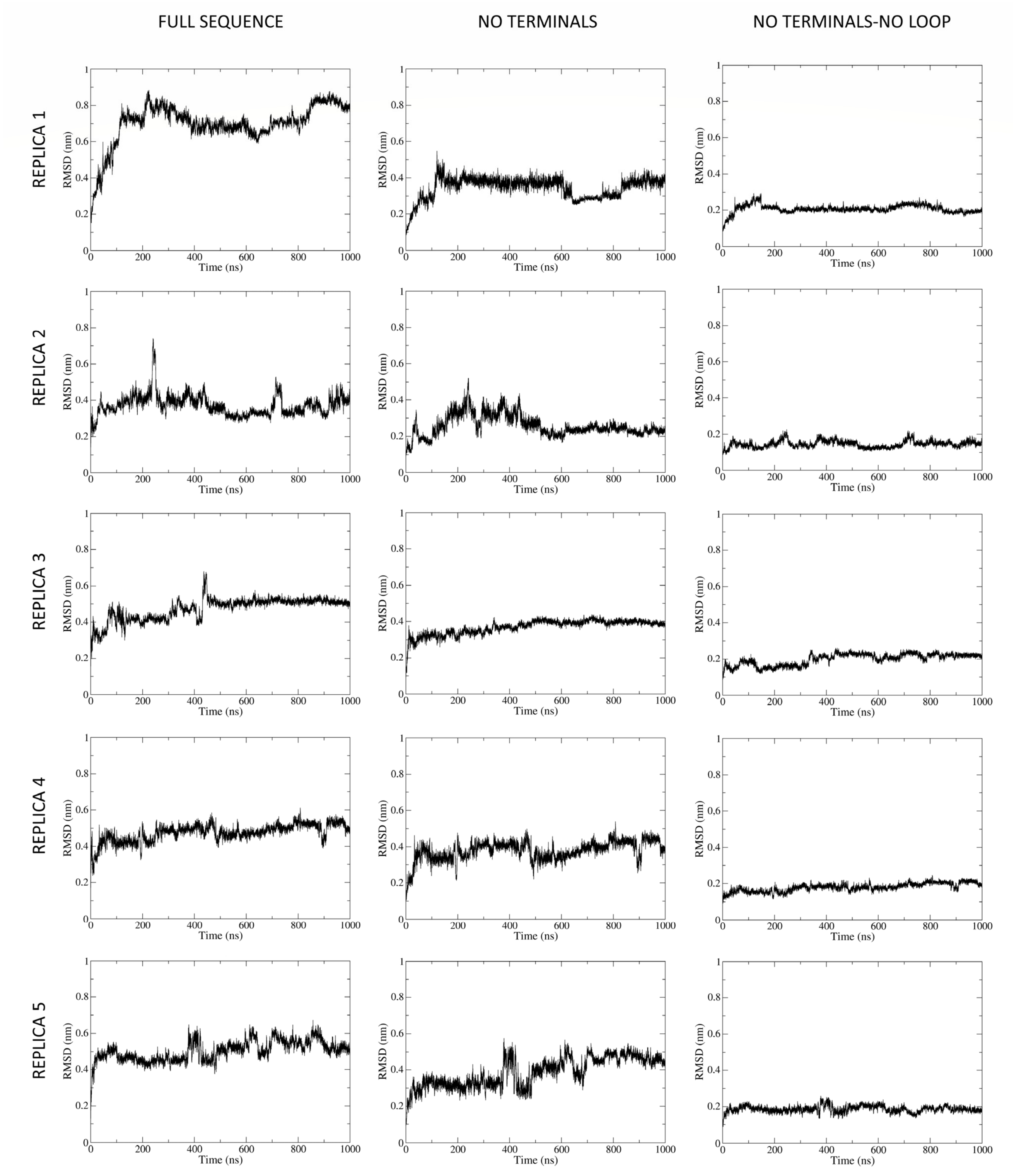

Figure S4. Protein RMSD of the MOE-completed LANCL2 (apo-form) along the 1 μs MD simulation. The RMSD was calculated on protein Cα and for the full sequence, removing the terminals, and removing terminals and the internal loop (34-60).

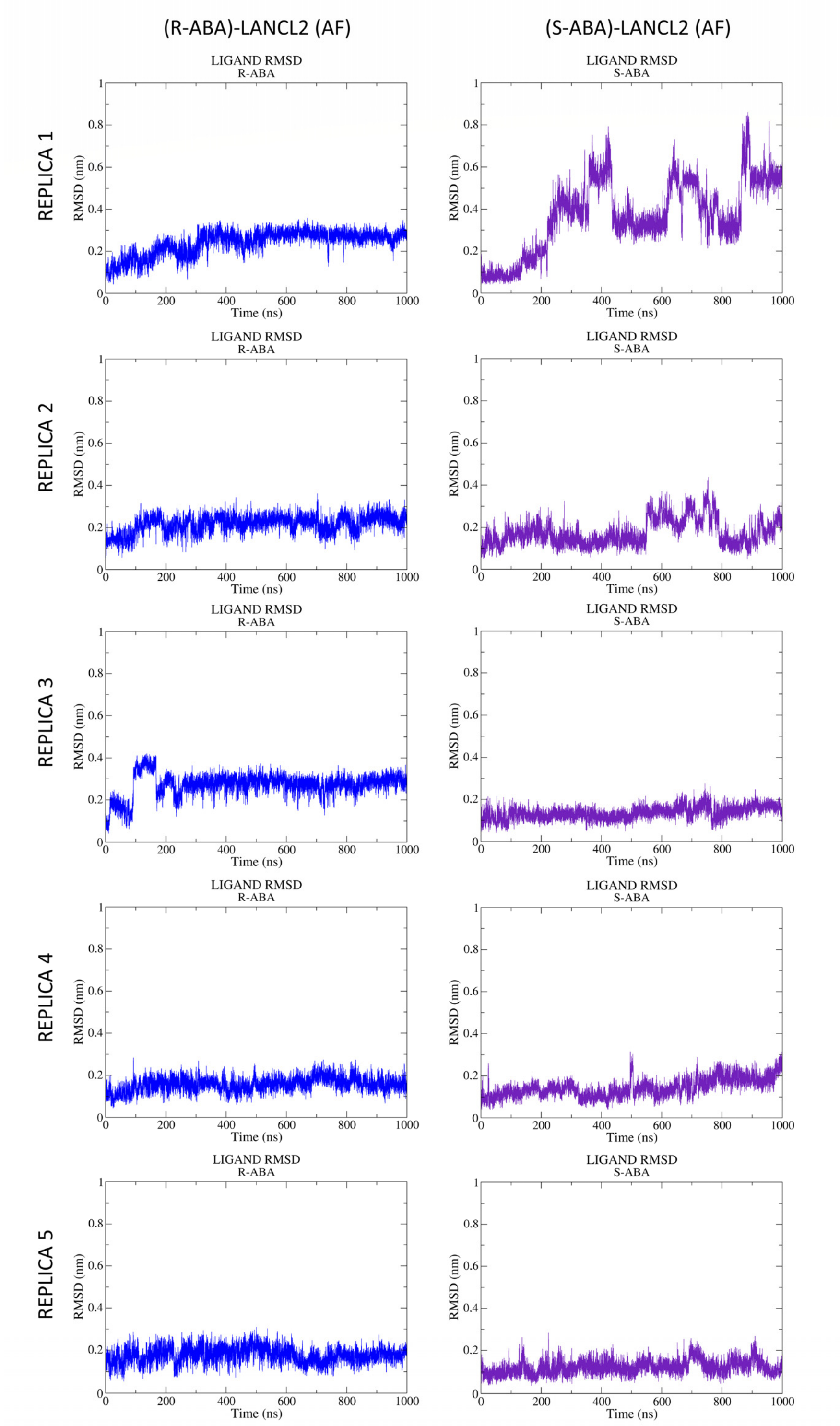

Figure S5. Ligand RMSD for (*R*/*S*)-**ABA**:LANCL2 (AF; MODEL 1) complex.

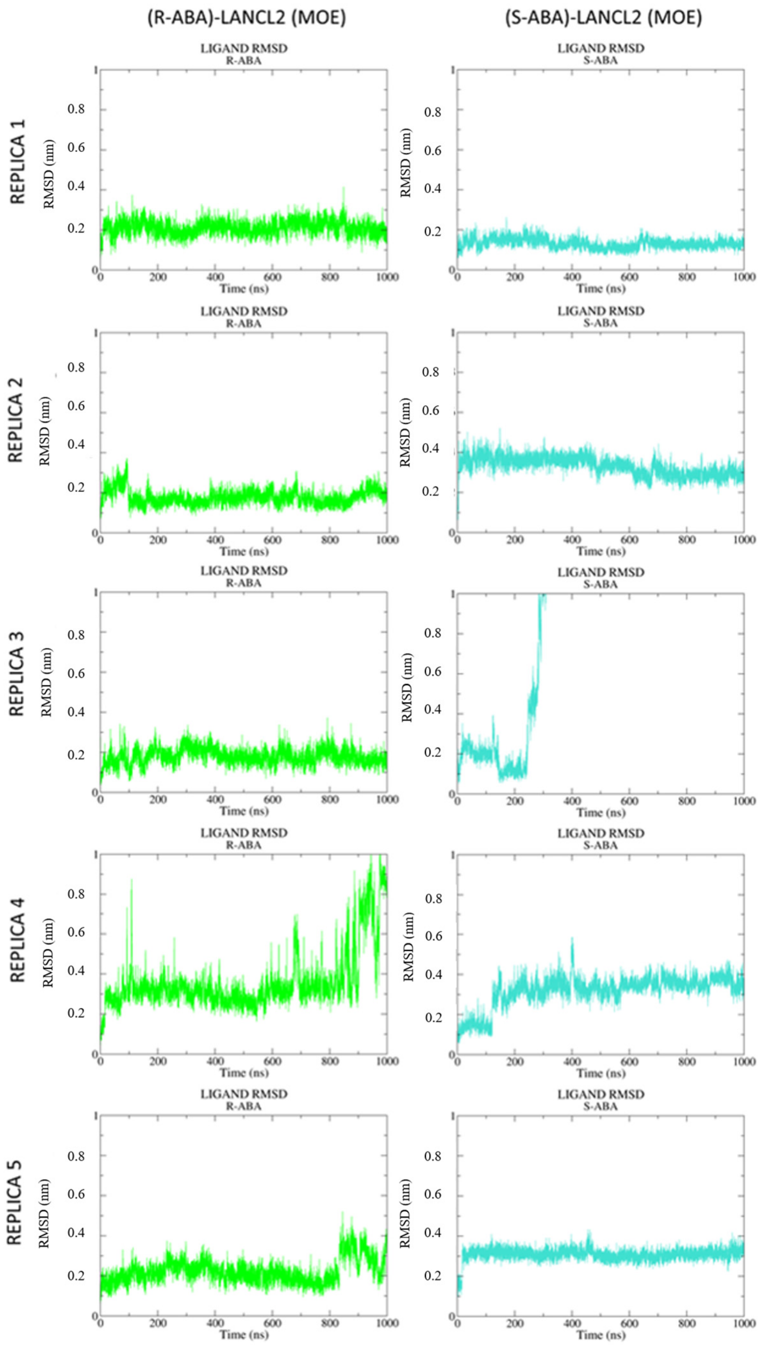

Figure S6. Ligand RMSD for (*R*/*S*)-**ABA**:LANCL2 complex (MOE model, MODEL 2). *R*-**ABA**: green, *S*-**ABA**: cyan.

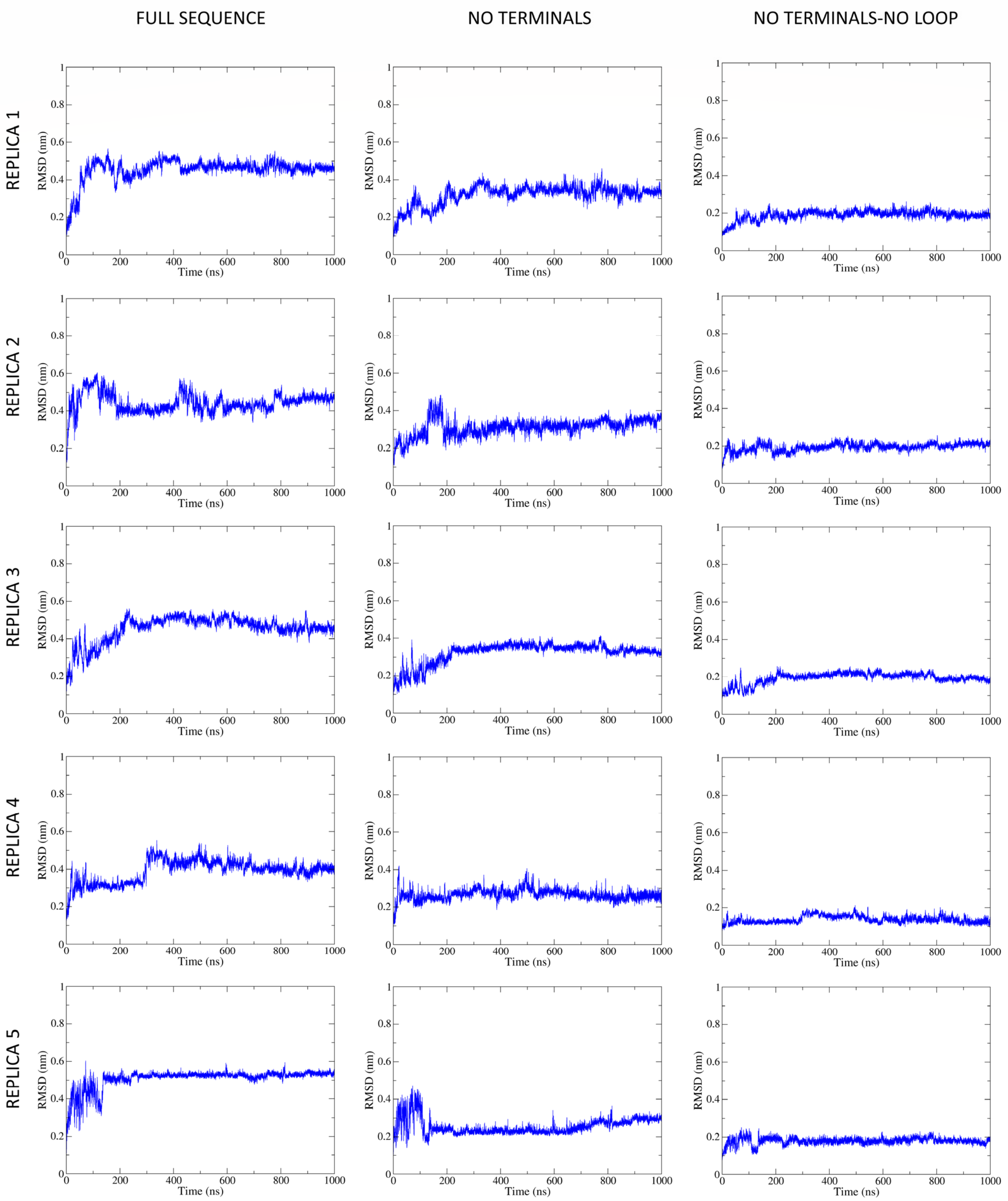

Figure S7. Protein RMSD of the AF-completed LANCL2 in complex with *R*-**ABA** along the 1 µs MD simulation. The RMSD was calculated on protein Cα and for the full sequence, removing the terminals, and removing terminals and the internal loop (34-60 residues).

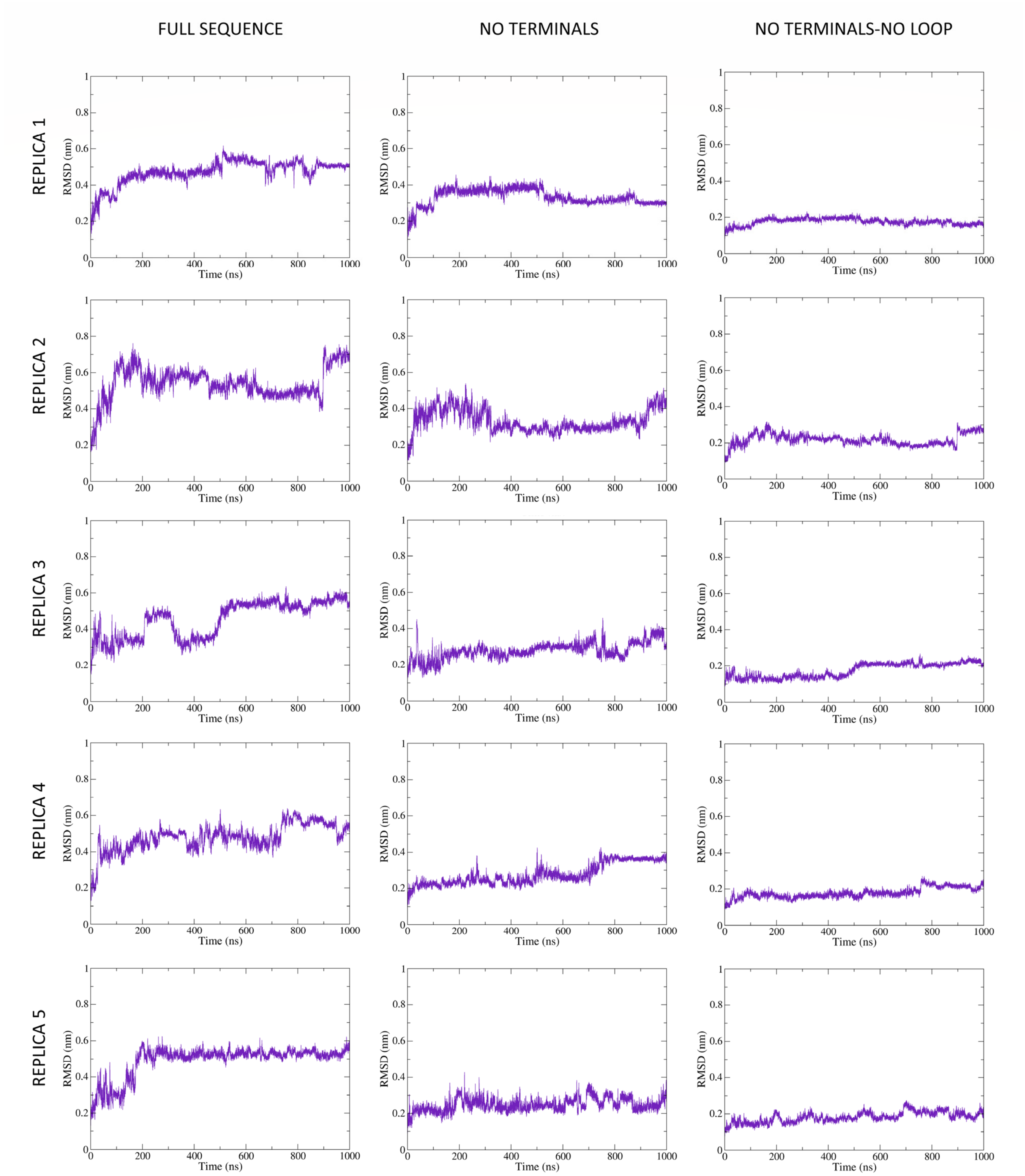

Figure S8. Protein RMSD of the AF-completed LANCL2 in complex with *S*-**ABA** along the 1 μs MD simulation. The RMSD was calculated on protein Cα and for the full sequence, removing the terminals, and removing terminals and the internal loop (34-60 residues).

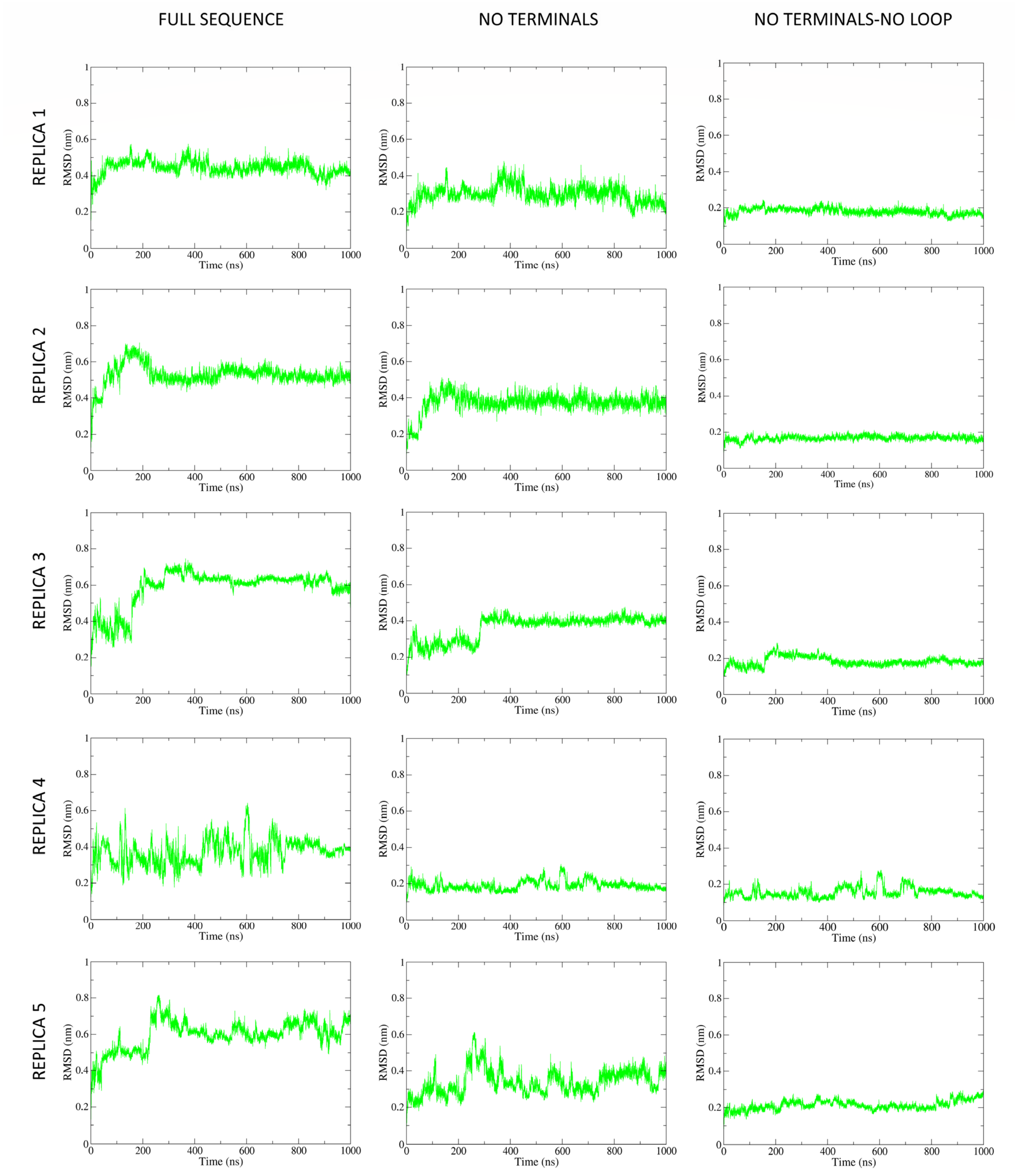

Figure S9. Protein RMSD of the MOE-completed LANCL2 in complex with *R*-**ABA** along the 1 µs MD simulation. The RMSD was calculated on protein Cα for the full sequence, removing the terminals, and removing terminals and the internal loop (34-60).

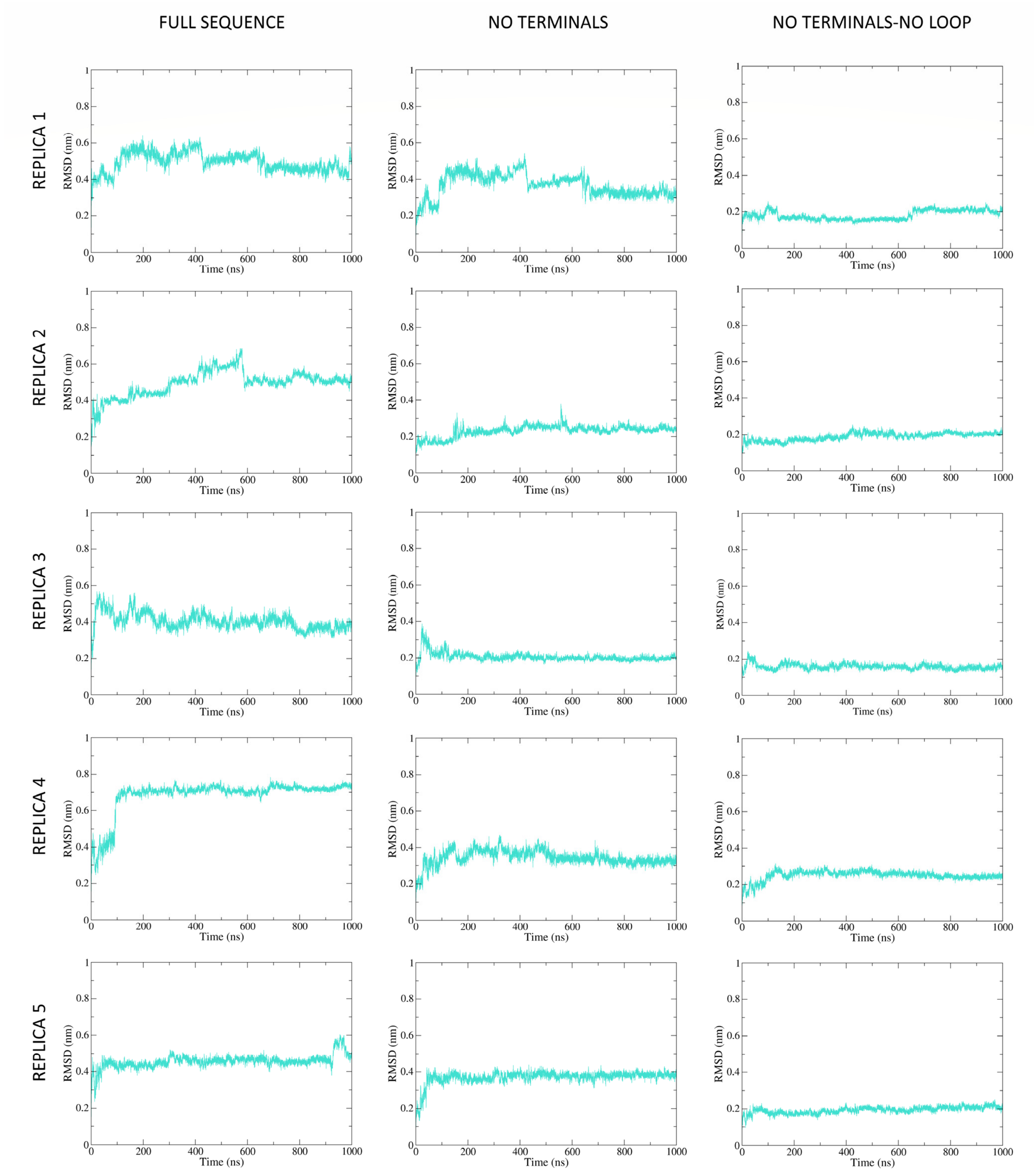

Figure S10. Protein RMSD of the MOE-completed LANCL2 in complex with *S*-**ABA** along the 1 µs MD simulation. The RMSD was calculated on protein Cα for the full sequence, removing the terminals, and removing terminals and the internal loop (34-60).

REPLICA 1	
Donor-Acceptor	Occupancy (%)
441ALA(H) - 902ABA(C=O)	91.5
438ARG(H21) - 902ABA(COO-)	39.3
118ARG(H21) - 902ABA(COO-)	10.6
118ARG(H11) - 902ABA(COO-)	13.8
118ARG(HE) - 902ABA(COO-)	29.3

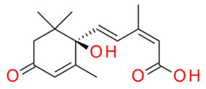

R -(-)-**ABA**

REPLICA 2	
Donor-Acceptor	Occupancy (%)
441ALA(H) - 902ABA(C=O)	90.0
438ARG(H21) - 902ABA(COO-)	34.4
118ARG(H21) - 902ABA(COO-)	25.6
118ARG(H11) - 902ABA(COO-)	42.1
118ARG(HE) - 902ABA(COO-)	19.3
164LYS(HZ1) - 902ABA(COO-)	11.8
438ARG(H21) - 902ABA(OH)	10.3
118ARG(H11) - 902ABA(OH)	15.9

REPLICA 3	
Donor-Acceptor	Occupancy (%)
441ALA(H) - 902ABA(C=O)	87.4
438ARG(H21) - 902ABA(COO-)	17.6
438ARG(H11) - 902ABA(COO-)	24.9
118ARG(H21) - 902ABA(COO-)	25.0
118ARG(HE) - 902ABA(COO-)	68.5
449ARG(H11) - 902ABA(COO-)	12.6

REPLICA 4	
Donor-Acceptor	Occupancy (%)
441ALA(H) - 902ABA(C=O)	90.4
438ARG(H21) - 902ABA(COO-)	42.4
118ARG(H11) - 902ABA(COO-)	58.9
118ARG(HE) - 902ABA(COO-)	21.1
164LYS(HZ1) - 902ABA(COO-)	13.9
118ARG(H11) - 902ABA(OH)	21.8
438ARG(H21) - 902ABA(OH)	13.3
438ARG(HE) - 902ABA(OH)	12.8

REPLICA 5	
Donor-Acceptor	Occupancy (%)
441ALA(H) - 902ABA(C=O)	91.0
438ARG(H21) - 902ABA(COO-)	28.2
118ARG(H11) - 902ABA(COO-)	49.1
118ARG(HE) - 902ABA(COO-)	24.1
164LYS(HZ1) - 902ABA(COO-)	19.2
438ARG(H21) - 902ABA(OH)	18.2
438ARG(HE) - 902ABA(OH)	14.8

Figure S11. H-bond contacts analysis of the *R*-**ABA**:LANCL2 (AF) MD simulations. The chemical structure of the ligand is reported.

REPLICA 1	
Donor-Acceptor	Occupancy (%)
441ALA(H) - 902ABA(C=O)	18.0
118ARG(H11) - 902ABA(COO-)	17.3
164LYS(HZ1) - 902ABA(COO-)	23.9
209TYR(HH) - 902ABA(C=O)	50.2
902ABA(OH) - 213GLU(OE2)	27.3
902ABA(OH) - 213GLU(OE1)	13.2

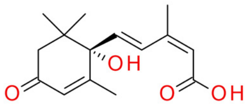

S -(+)-**ABA**

REPLICA 2	
Donor-Acceptor	Occupancy (%)
441ALA(H) - 902ABA(C=O)	73.2
118ARG(H11) - 902ABA(COO-)	23.9
118ARG(HE) - 902ABA(COO-)	22.3
449ARG(H11) - 902ABA(COO-)	23.4
164LYS(HZ1) - 902ABA(COO-)	13.8
209TYR(HH) - 902ABA(C=O)	21.4
118ARG(H11) - 902ABA(OH)	23.0

REPLICA 3	
Donor-Acceptor	Occupancy (%)
441ALA(H) - 902ABA(C=O)	94.7
118ARG(H21) - 902ABA(COO-)	23.2
118ARG(H11) - 902ABA(COO-)	49.4
164LYS(HZ1) - 902ABA(COO-)	36.5
902ABA(OH) - 213GLU(OE2)	79.9
438ARG(H11) - 902ABA(OH)	10.2
438ARG(HE) - 902ABA(OH)	18.4

REPLICA 4	
Donor-Acceptor	Occupancy (%)
441ALA(H) - 902ABA(C=O)	90.6
438ARG(H21) - 902ABA(COO-)	46.6
438ARG(H11) - 902ABA(COO-)	16.6
118ARG(H11) - 902ABA(COO-)	37.9
164LYS(HZ1) - 902ABA(COO-)	21.4
902ABA(OH) - 213GLU(OE1)	13.9
438ARG(HE) - 902ABA(OH)	23.0
438ARG(H21) - 902ABA(OH)	13.4

REPLICA 5	
Donor-Acceptor	Occupancy (%)
441ALA(H) - 902ABA(C=O)	93.9
438ARG(H11) - 902ABA(COO-)	16.6
164LYS(HZ1) - 902ABA(COO-)	15.1
118ARG(H11) - 902ABA(COO-)	53.3
438ARG(H11) - 902ABA(OH)	38.4
438ARG(HE) - 902ABA(OH)	12.5

Figure S12. H-bond contacts analysis of the *S*-**ABA**:LANCL2 (AF) MD simulations. The chemical structure of the ligand is reported.

REPLICA 1	
Donor-Acceptor	Occupancy (%)
441ALA(H) - 902ABA(C=O)	93.6
118ARG(H11) - 902ABA(COO-)	57.9
118ARG(HE) - 902ABA(COO-)	28.3
164LYS(HZ1) - 902ABA(COO-)	24.5
118ARG(H11) - 902ABA(OH)	13.2

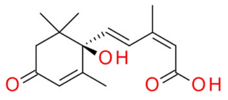

R -(-)-**ABA**

REPLICA 2	
Donor-Acceptor	Occupancy (%)
441ALA(H) - 902ABA(C=O)	92.6
118ARG(HE) - 902ABA(COO-)	60.3
438ARG(H21) - 902ABA(COO-)	12.6
438ARG(H11) - 902ABA(COO-)	40.4
164LYS(HZ1) - 902ABA(COO-)	12.1
438ARG(H11) - 902ABA(OH)	38.0

REPLICA 3	
Donor-Acceptor	Occupancy (%)
441ALA(H) - 902ABA(C=O)	91.6
118ARG(H11) - 902ABA(COO-)	79.2
438ARG(H21) - 902ABA(COO-)	35.7
438ARG(H11) - 902ABA(COO-)	15.0
164LYS(HZ1) - 902ABA(COO-)	18.5
118ARG(H11) - 902ABA(OH)	13.1

REPLICA 4	
Donor-Acceptor	Occupancy (%)
449ARG(H21) - 902ABA(COO-)	60.8
449ARG(HE) - 902ABA(COO-)	21.1
209TYR(HH) - 902ABA(C=O)	89.5

REPLICA 5	
Donor-Acceptor	Occupancy (%)
441ALA(H) - 902ABA(C=O)	77.7
118ARG(H11) - 902ABA(COO-)	59.1
118ARG(HE) - 902ABA(COO-)	17.8
164LYS(HZ1) - 902ABA(COO-)	23.9
209TYR(HH) - 902ABA(C=O)	17.1

Figure S13. H-bond contacts analysis of the *R*-**ABA**:LANCL2 (MOE) MD simulations. The chemical structure of the ligand is reported.

REPLICA 1	
Donor-Acceptor	Occupancy (%)
441ALA(H) - 902ABA(C=O)	95.6
438ARG(H21) - 902ABA(COO-)	27.9
118ARG(H21) - 902ABA(COO-)	15.0
118ARG(H11) - 902ABA(COO-)	14.5
118ARG(HE) - 902ABA(COO-)	52.9
449ARG(H21) - 902ABA(COO-)	15.3
448LYS(HZ1) - 902ABA(COO-)	23.7

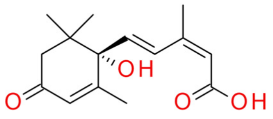

S -(+)-**ABA**

REPLICA 2	
Donor-Acceptor	Occupancy (%)
118ARG(H11) - 902ABA(COO-)	14.9
449ARG(H21) - 902ABA(COO-)	63.4
449ARG(H21) - 902ABA(COO-)	25.9
449ARG(H11) - 902ABA(COO-)	14.9
449ARG(H11) - 902ABA(COO-)	23.7
449ARG(HE) - 902ABA(COO-)	43.8
209TYR(HH) - 902ABA(C=O)	98.0
447SER(HG) - 902ABA(COO-)	13.2

REPLICA 3	
Donor-Acceptor	Occupancy (%)
441ALA(H) - 902ABA(C=O)	10.1
438ARG(H11) - 902ABA(COO-)	13.1
125TYR(HH) - 902ABA(COO-)	10.8
82LYS(HZ1) - 902ABA(COO-)	12.6
78GLN(E21) - 902ABA(COO-)	17.5
438ARG(H11) - 902ABA(OH)	10.2

REPLICA 4	
Donor-Acceptor	Occupancy (%)
441ALA(H) - 902ABA(C=O)	11.7
438ARG(H21) - 902ABA(COO-)	10.0
438ARG(H11) - 902ABA(COO-)	33.4
209TYR(HH) - 902ABA(C=O)	47.3
438ARG(H11) - 902ABA(OH)	36.2

REPLICA 5	
donor-acceptor	Occupancy (%)
449ARG(H21) - 902ABA(COO-)	87.4
449ARG(H11) - 902ABA(COO-)	59.7
438ARG(H11) - 902ABA(COO-)	92.9
209TYR(HH) - 902ABA(C=O)	97.7
438ARG(H11) - 902ABA(OH)	95.4

Figure S14. H-bond contacts analysis of the *S*-**ABA**:LANCL2 (MOE) MD simulations. The chemical structure of the ligand is reported.

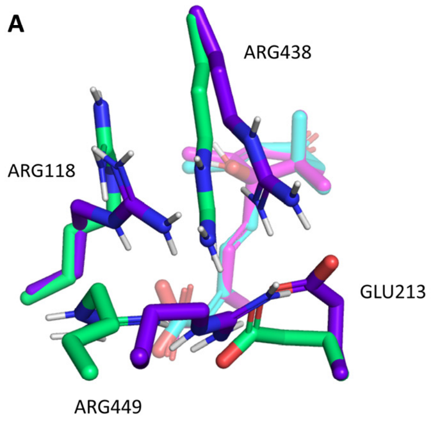

Figure S15. Comparison between the starting complexes of (*R*/*S*)-**ABA** in the two models. (A) *R*-**ABA** complex with MOE (purple/hotpink) and AF (green/cyan) models. (B) *S*-**ABA** in complex with MOE (green/cyan) and AF (purple/hotpink) models.

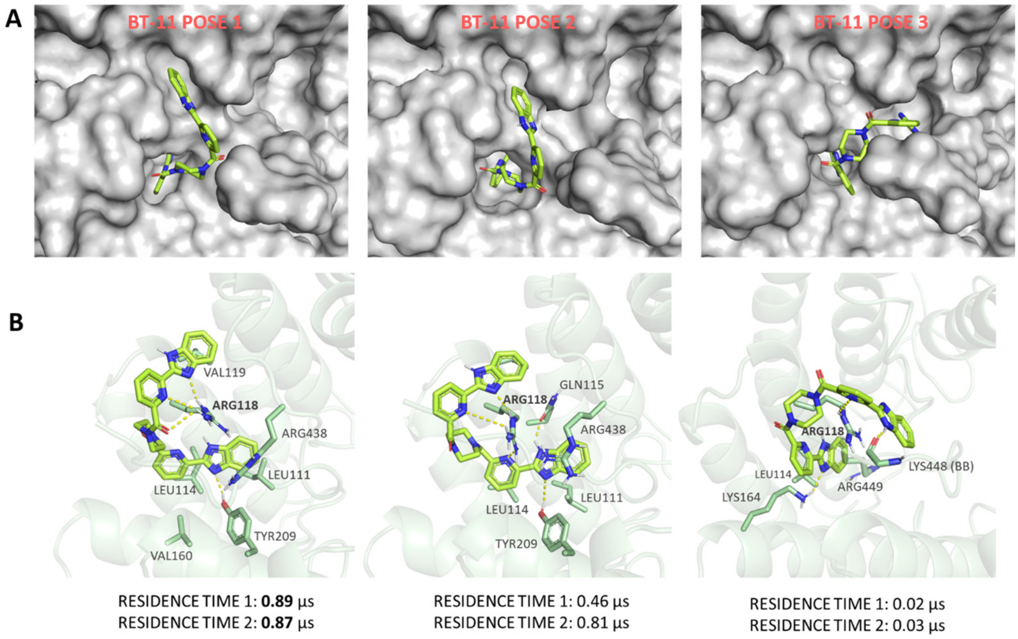

Figure S16. Three **BT-11** docking poses were selected via molecular docking at the LANCL2 site 1. (A). Residence times of the preliminary MDs are reported (B).

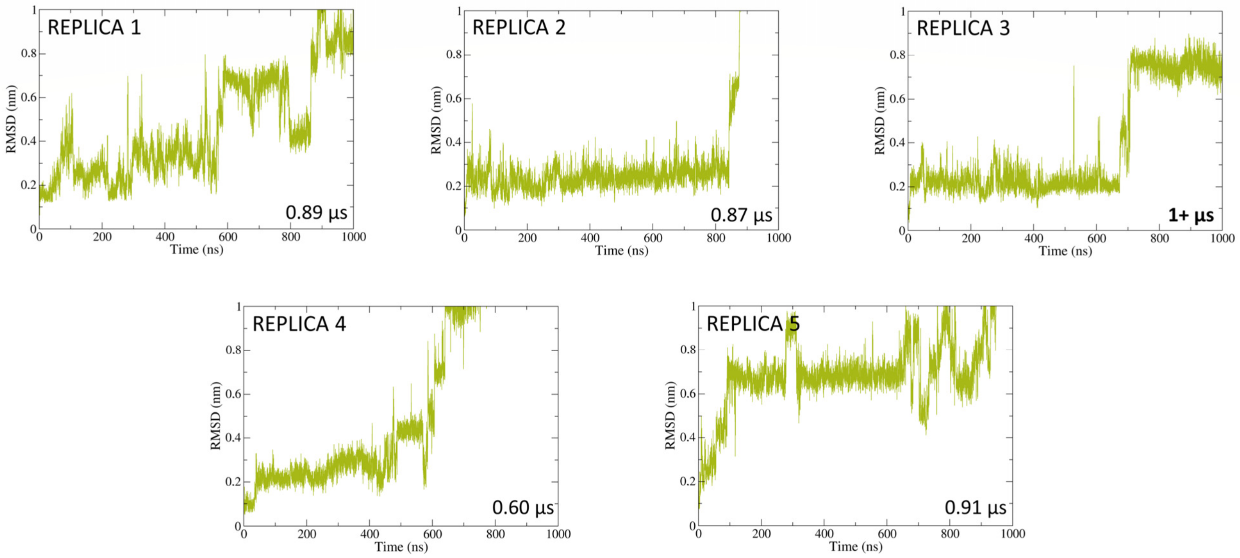

Figure S17. Ligand RMSD and residence time for **BT-11**/LANCL2 complexes.

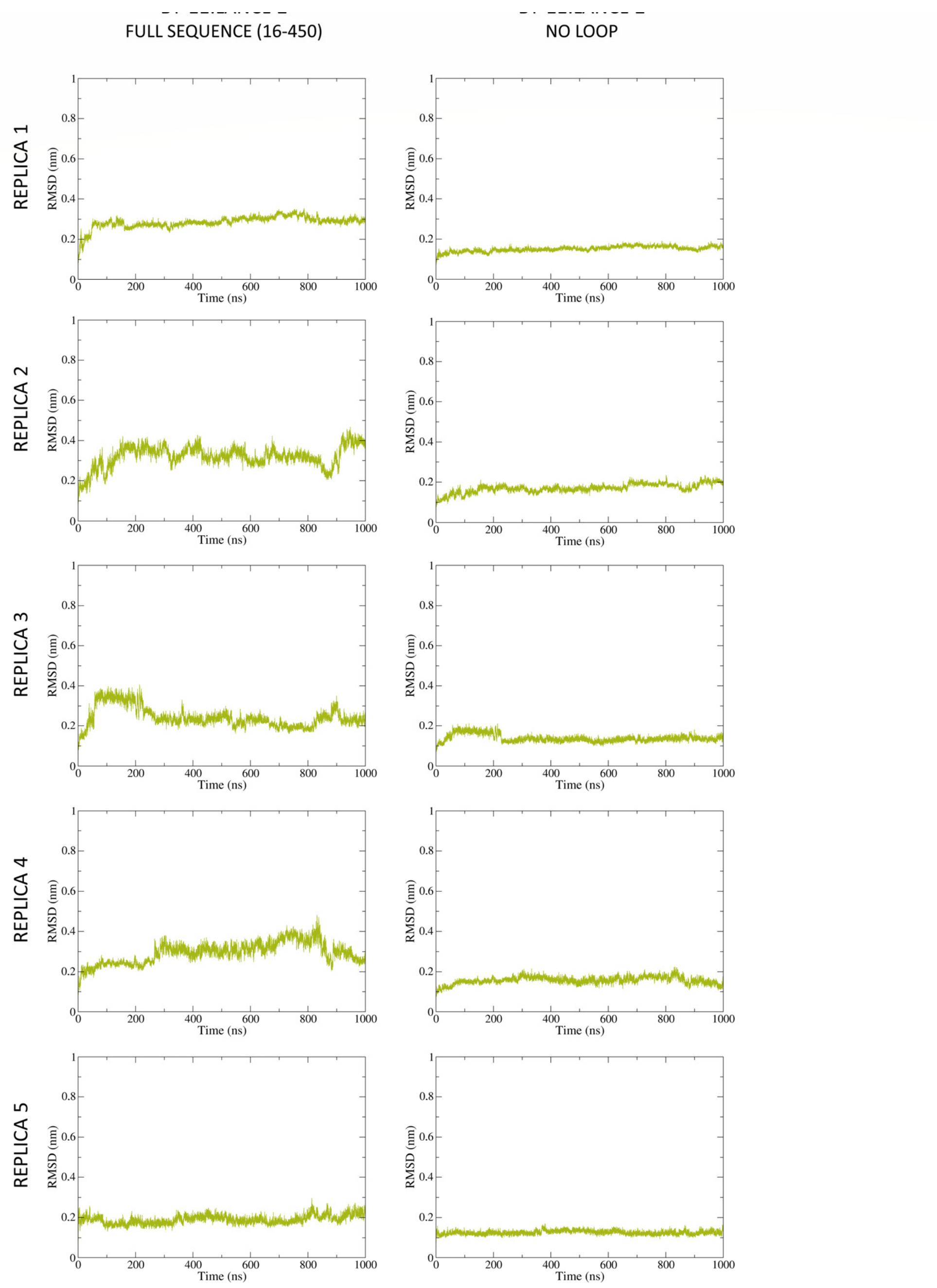

Figure S18. Protein RMSD of LANCL2 in complex with **BT-11** (docking pose 1) along the 1 μs MD simulation. The RMSD was calculated on protein Cα for the sequence 16-450, and after the removal of the internal loop (34-60). The removal of terminals was not necessary, as the model used for **BT-11** MDs was missing part of the N-terminus (residue 1-16), which is responsible for the majority of the movements of the terminals. The C-terminus was instead kept as in the AlphaFold-completed model used for ABA simulations.

REPLICA 1	
donor-acceptor	Occupancy (%)
450ASP(H) - 887BT1(O)	14.8
449ARG(H) - 887BT1(O)	51.7
209TYR(HH) - 887BT1(N6)	49.5
164LYS(HZ1) - 887BT1(O1)	11.2
887BT1(H13) - 114LEU(O)	17.1

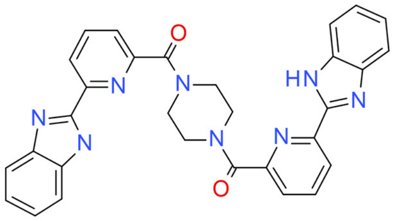

BT-11

REPLICA 2	
donor-acceptor	Occupancy (%)
209TYR(HH) - 887BT1(N6)	12.2
118ARG(H21) - 887BT1(O)	12.6
118ARG(HE) - 887BT1(O)	19.1
887BT1(H6) - 436THR(OG1)	13.3
887BT1(H6) - 118ARG(NH2)	12.1

REPLICA 4	
donor-acceptor	Occupancy (%)
209TYR(HH) - 887BT1(N6)	76.1
118ARG(HE) - 887BT1(N2)	26.8

REPLICA 3	
donor-acceptor	Occupancy (%)
887BT1(H13) - 213GLU(OE2)	42.9
887BT1(H13) - 213GLU(OE1)	19.2

REPLICA 5	
donor-acceptor	Occupancy (%)
450ASP(H) - 887BT1(O1)	12.5
164LYS(HZ1) - 887BT1(O1)	43.0

Figure S19. H-bond contacts analysis of the **BT-11:**LANCL2 (AF) MD simulations. The chemical structure of the ligand is reported.

H-bonds for the **BT11**:LANCL2 complex					
REPLICA N.	ARG 449	TYR 209	GLU 213	ARG 118	LYS 164
1	YES (52%)	YES (49%)	NO	NO	NO
2	NO	NO	NO	NO	NO
3	NO	NO	YES (43%)	NO	NO
4	NO	YES (76%)	NO	YES (27%)	NO
5	NO	NO	NO	NO	YES (43%)

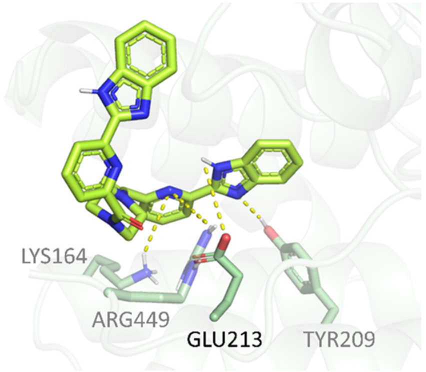

Figure S20. BT-11 H-bond analysis table and representation. Highlighted residues are from stable replica 3, transparent residues establish H-bonds with the ligand in non-stable replicas.

Table S1. *R*-**ABA** docking scores as obtained via (SITE1)-IF protocol at the Alphafold (AF)-completed model (MODEL 1). The compound pose retained for the following computational analyses is highlighted in light violet.

POSE N.	docking score	glide gscore	IFDScore
1	-8.899	-8.899	-892.86
2	-7.776	-7.776	-892.59
3	-7.175	-7.175	-892.21

4	-7.780	-7.780	-892.08
5	-7.846	-7.846	-892.01

Table S2. *S*-**ABA** docking scores as obtained via (SITE1)-IF protocol at the Alphafold (AF)-completed model (MODEL 1). The compound pose retained for the following computational analyses is highlighted in light violet.

POSE N.	docking score	glide gscore	IFDScore
1	-7.977	-7.977	-894.88
2	-8.036	-8.036	-894.62
3	-7.750	-7.750	-894.04
4	-8.341	-8.341	-893.82
5	-7.125	-7.125	-893.50

Table S3. *R*-**ABA** docking scores as obtained via (SITE1)-IF protocol at the MOE-completed model (MODEL 2). The compound pose retained for the following computational analyses is highlighted in light violet.

POSE N.	docking score	glide gscore	IFDScore
1	-8.432	-8.432	-912.11
2	-8.331	-8.331	-911.81
3	-9.290	-9.290	-911.69
4	-7.879	-7.879	-910.87
5	-7.846	-7.846	-910.33

Table S4. *S*-**ABA** docking scores as obtained via (SITE1)-IF protocol at the MOE-completed model (MODEL 2). The compound pose retained for the following computational analyses is highlighted in light violet.

POSE N.	docking score	glide gscore	IFDScore
1	-8.646	-8.646	-880.03
2	-8.469	-8.469	-878.78
3	-8.033	-8.033	-878.43
4	-7.872	-7.872	-878.31
5	-7.287	-7.287	-878.22

Table S5. BT-11 docking scores as obtained via (SITE1)-IF protocol at the Alphafold (AF)-completed model (MODEL 1). The compound poses retained for the following computational analyses are highlighted in light violet.

Pose n.	docking score	glide gscore	IFDScore
**1**	-8.197	-8.197	-896.67
**2**	-8.942	-8.942	-896.32
3	-7.953	-7.953	-896.05
4	-6.868	-6.868	-894.23
**5**	-6.652	-6.652	-893.85

Table S6. Description of the three performed MD equilibrations.

Equilibration n.	Time (ns)	Ensemble	Heavy atoms positional restraints (module kJ/mol)
1	0.5	NVT	Yes (1000)
2	0.5	NPT	Yes (1000)
3	1	NPT	No

Table S7. Primer sequences used to amplify rat target genes.

Rat genes	Accession N.	Forward Primer 5′-3′	Reverse Primer 5′-3′
Prkaa2	NM_019142	AGAAGCAGAAGCACGACGG	GAAGGTGCCGACGCCC
Ppargc1a	NM_031347	GCACACATCGCAATTCTCCC	CTCTGCGGTATTCGTCCCTC
Sirt1	NM_001372090	CAGTGTCATGGTTCCTTTGC	CACCGAGGAACTACCTGA T
Nos3	NM_021838	AGGCCTTGGTATTGGTGGTG	TAGGGGCCCGACATTTCC AT
Sod2	NM_017051	TAAGGGTGGTGGAGAACCCA	ACCTTGGACTCCCACAGACA
Gpx4	NM_017165	CCGTCTGAGCCGCTTATTGA	AATCATCGCGGGATGCACA
Hprt1	NM_012583	TTGGTCAAGCAGTACAGCCC	TGGCCTGTATCCAACACTTCG

Table S8. Apo-LANCL2 (AlphaFold model; MODEL 1) PCA analysis. The main movements individuated by the principal components (PCs) are represented as the superposition of 50 states, going from dark to light colors. Two PCs were considered. For each PC, front and back perspective images are reported.

REPLICA N.	PC1 (FRONT AND BACK)	PC2 (FRONT AND BACK)
1	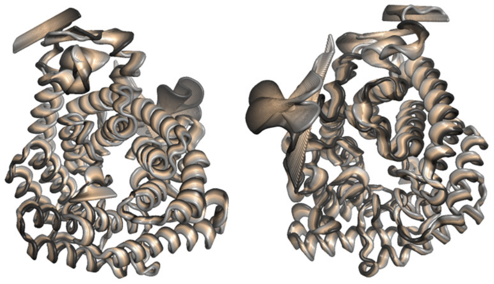	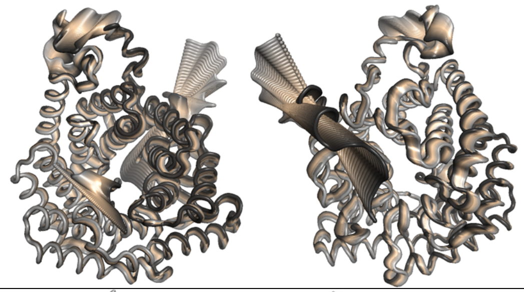
2	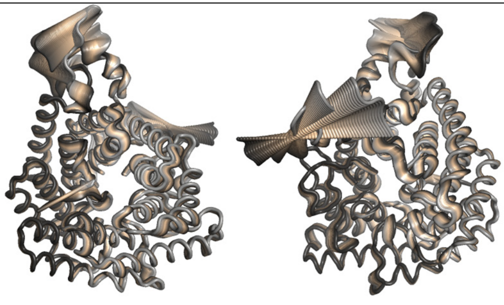	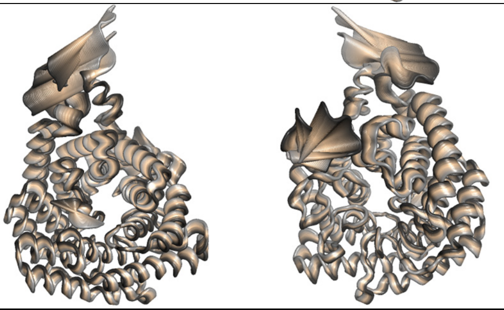
3	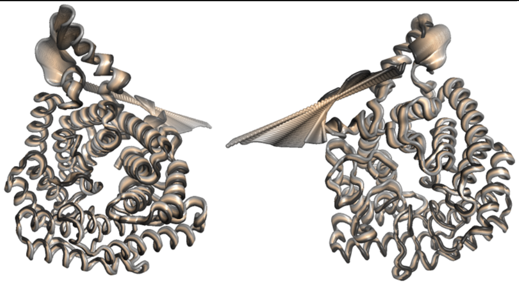	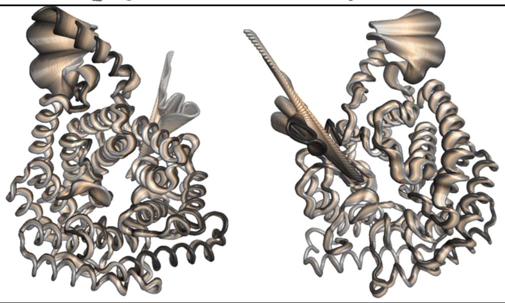
4	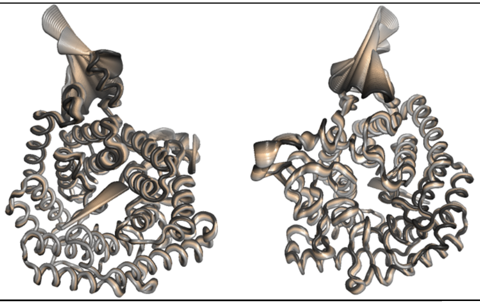	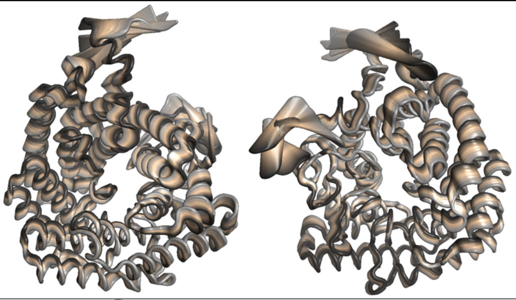
5	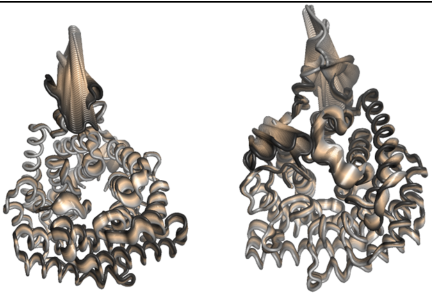	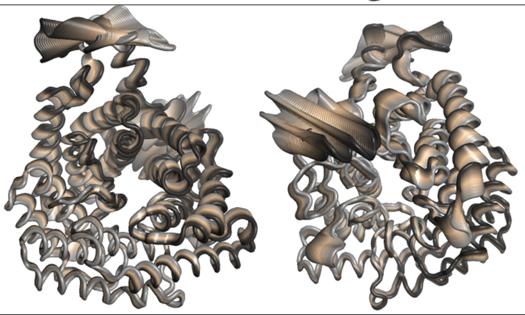

Table S9. Apo-LANCL2 (MOE model; MODEL 2) PCA analysis. The main movements individuated by the principal components (PCs) are represented as the superposition of 50 states, going from dark to light colors. Two PCs were considered. For each PC, front and back perspective images are reported.

REPLICA N.	PC1 (FRONT AND BACK)	PC2 (FRONT AND BACK)

1	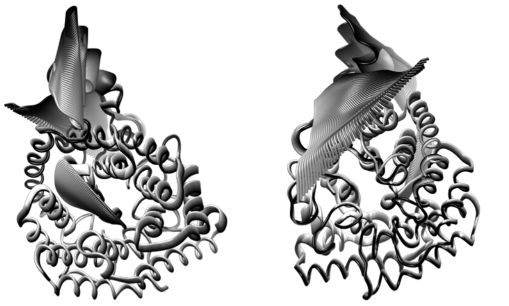	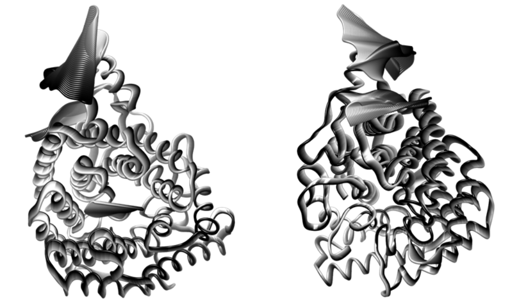
2	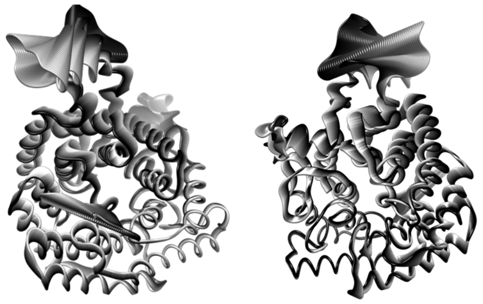	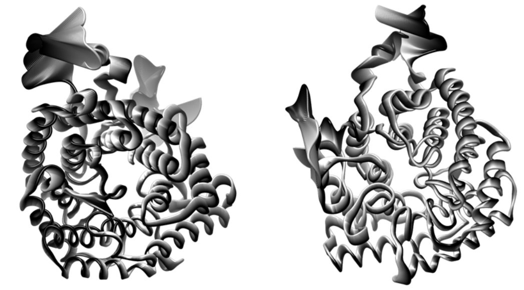
3	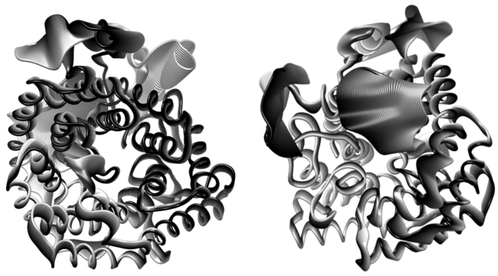	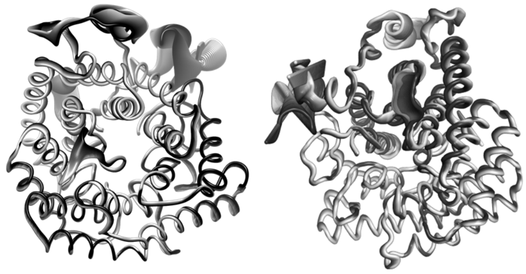
4	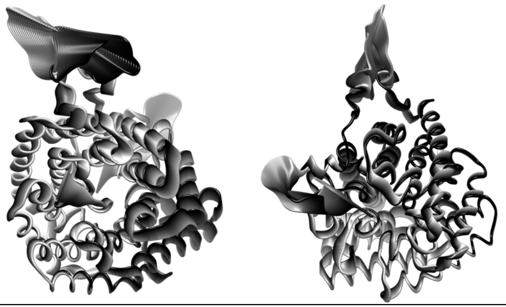	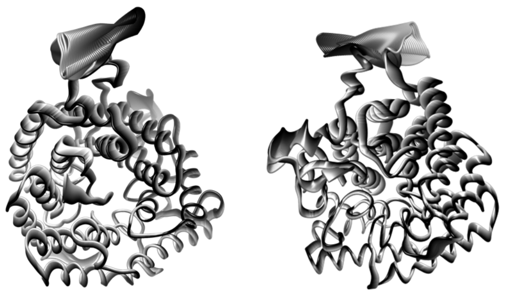
5	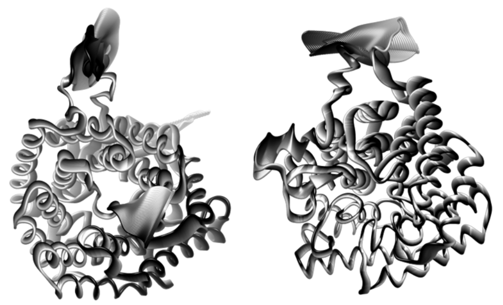	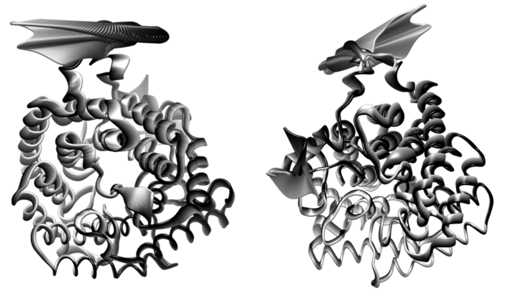

Table S10. *R*-**ABA**:LANCL2 (AlphaFold model) PCA analysis. The main movements individuated by the principal components (PCs) are represented as the superposition of 50 states, going from dark to light colors. Two PCs were considered. For each PC, front and back perspective images are reported.

REPLICA N.	PC1 (FRONT AND BACK)	PC2 (FRONT AND BACK)

1	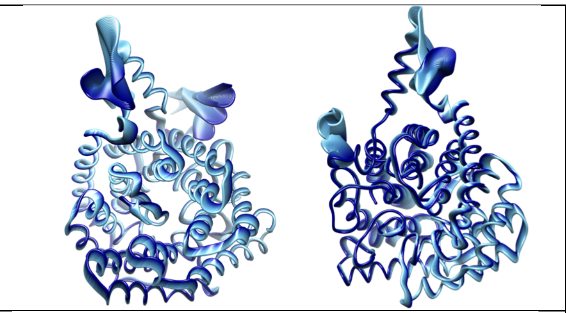	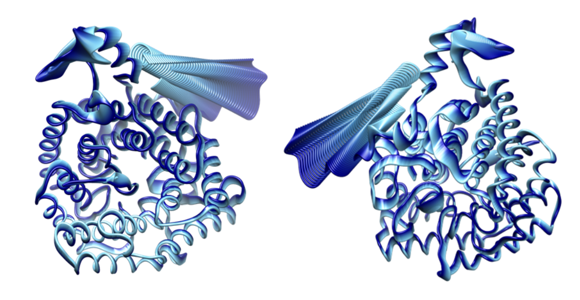
2	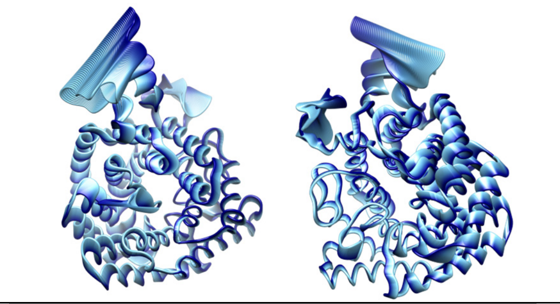	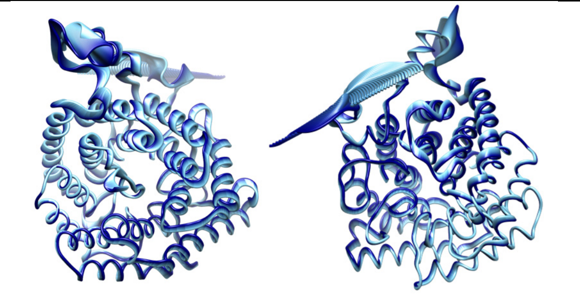
3	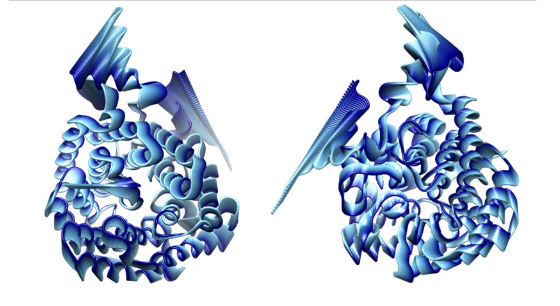	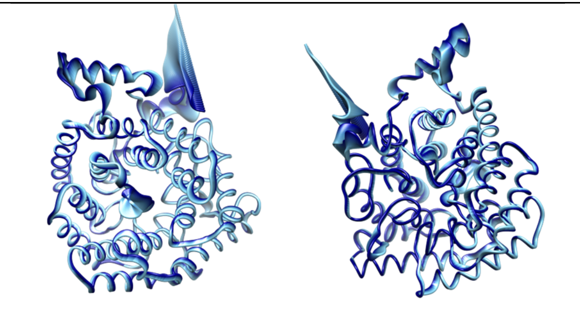
4	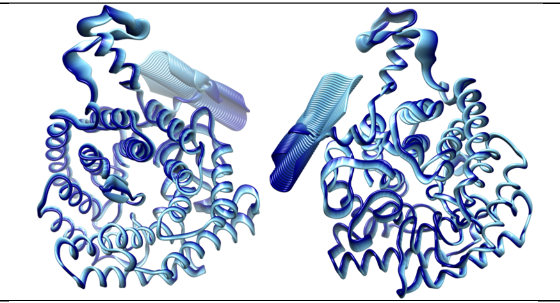	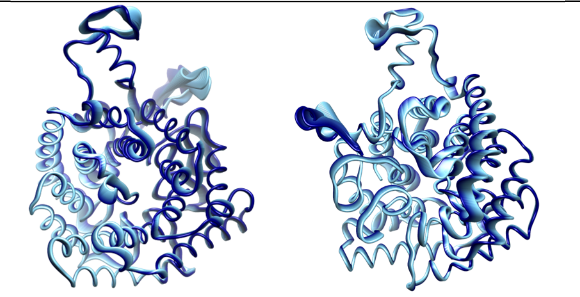
5	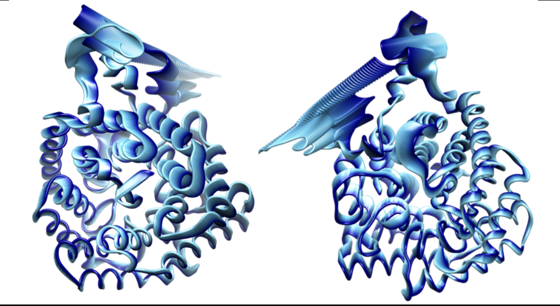	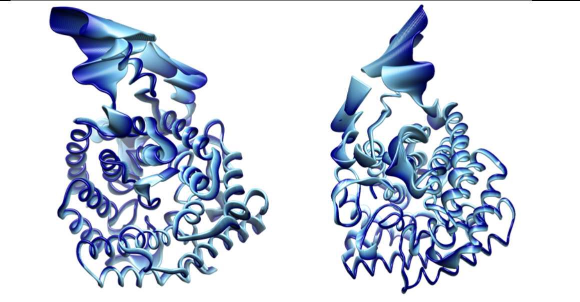

Table S11. *S*-**ABA**:LANCL2 (AlphaFold model) PCA analysis. The main movements individuated by the principal components (PCs) are represented as the superposition of 50 states, going from dark to light colours. Two PCs were considered. For each PC, front and back perspective images are reported.

REPLICA N.	PC1 (FRONT AND BACK)	PC2 (FRONT AND BACK)

Tables S12. *R*-**ABA**:LANCL2 (MOE model) PCA analysis. The main movements individuated by the principal components (PCs) are represented as the superposition of 50 states, going from dark to light colors. Two PCs were considered. For each PC, front and back perspective images are reported.

REPLICA N.	PC1 (FRONT AND BACK)	PC2 (FRONT AND BACK)
1	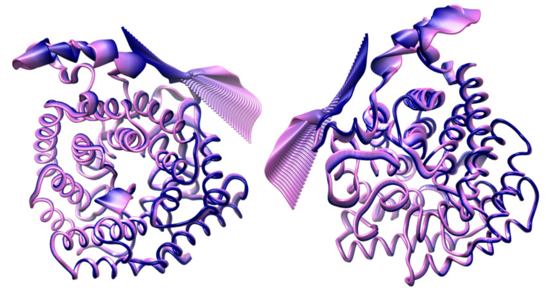	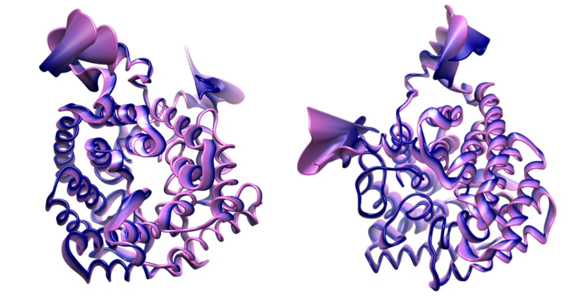
2	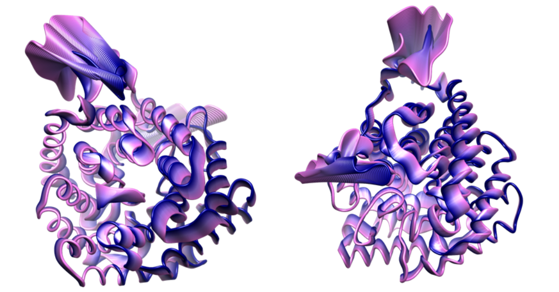	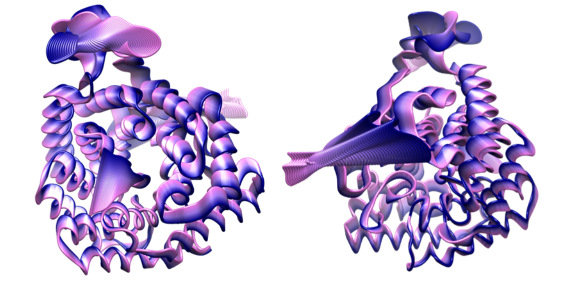
3	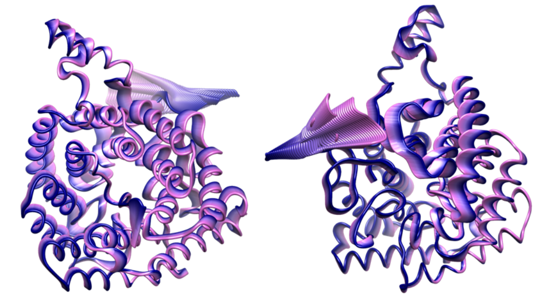	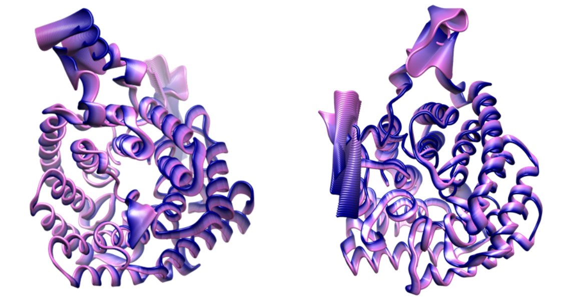
4	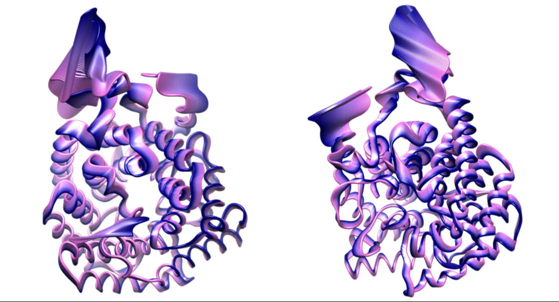	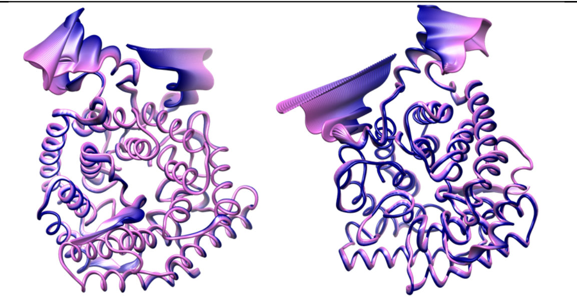
5	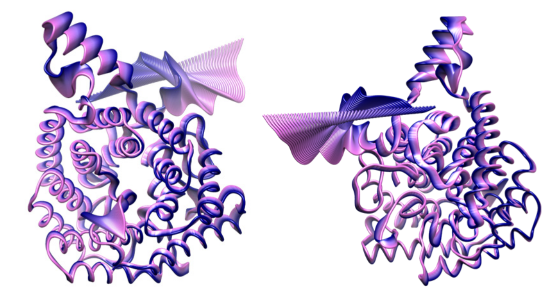	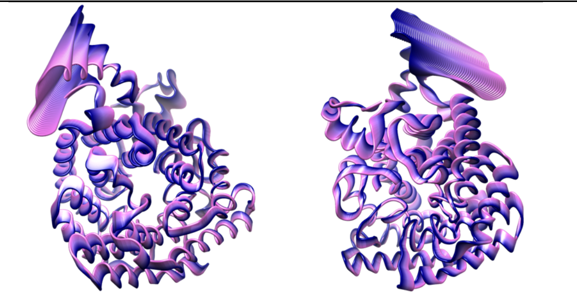

1	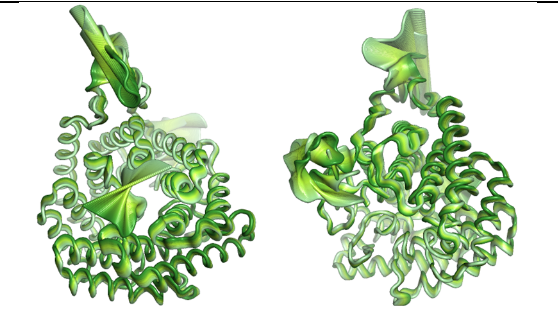	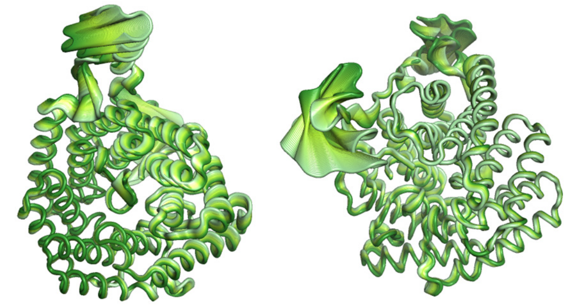
2	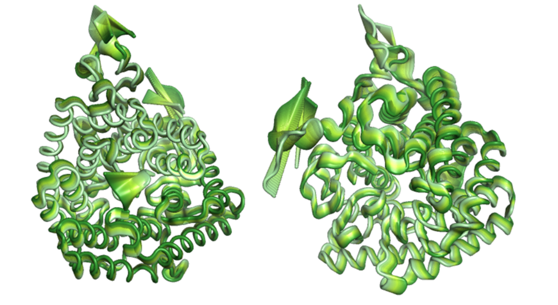	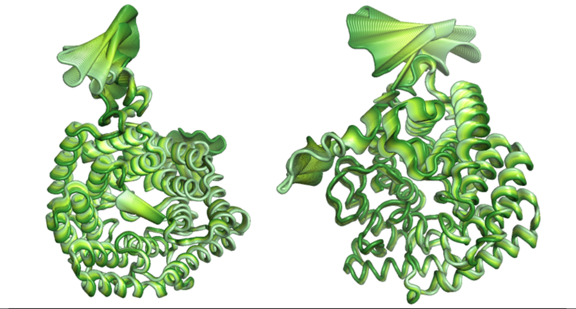
3	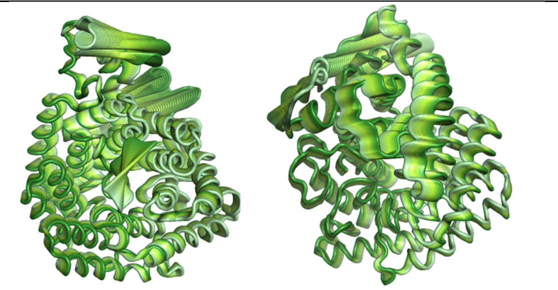	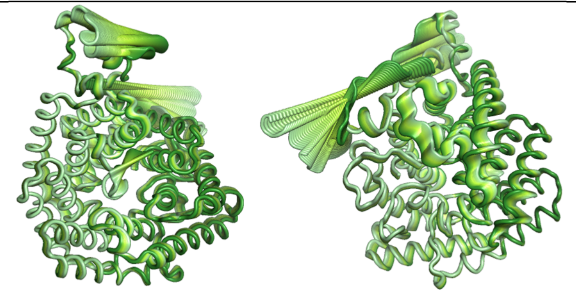
4	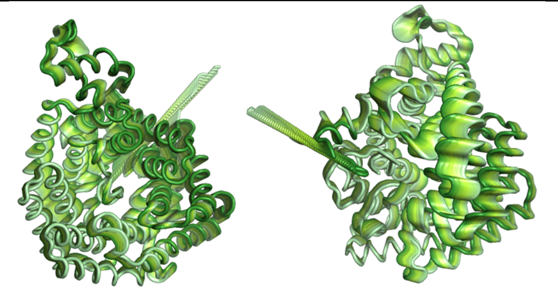	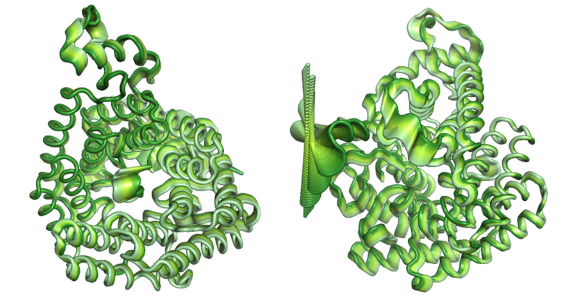
5	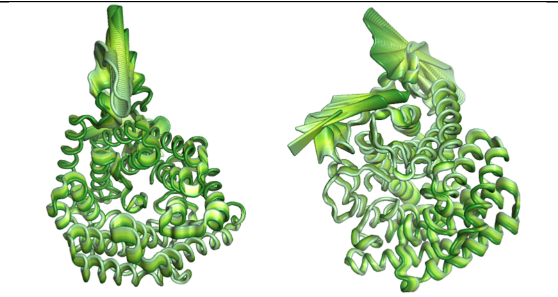	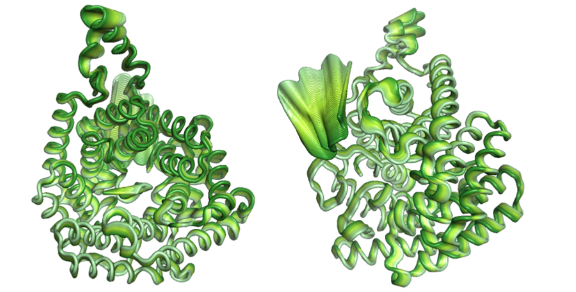

Table S13. *S*-**ABA**:LANCL2 (MOE model) PCA analysis. The main movements individuated by the principal components (PCs) are represented as the superposition of 50 states, going from dark to light colours. Two PCs were considered. For each PC, front and back perspective images are reported.

REPLICA N.	PC1 (FRONT AND BACK)	PC2 (FRONT AND BACK)
1	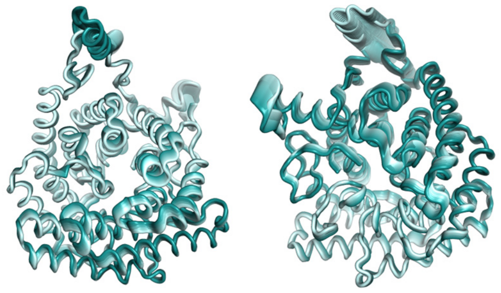	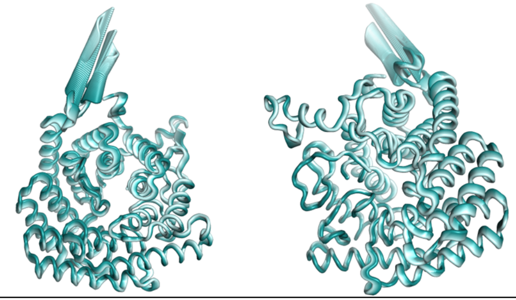
2	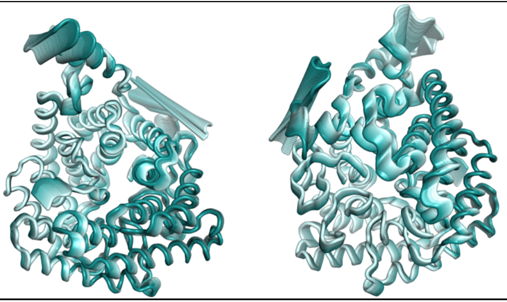	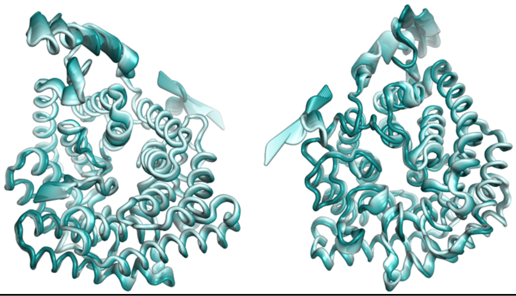
3	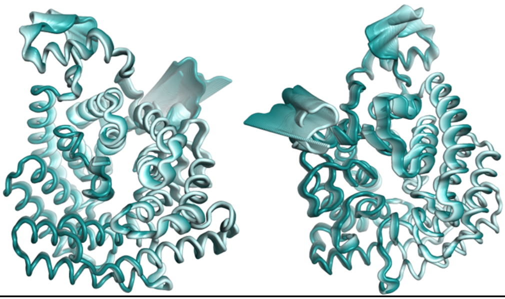	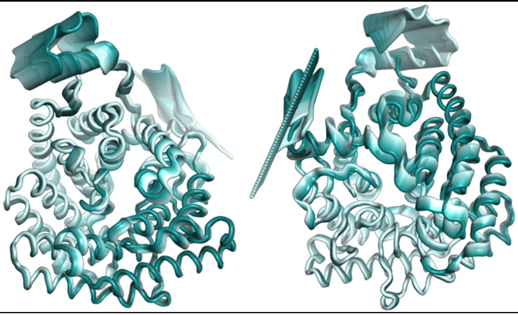
4	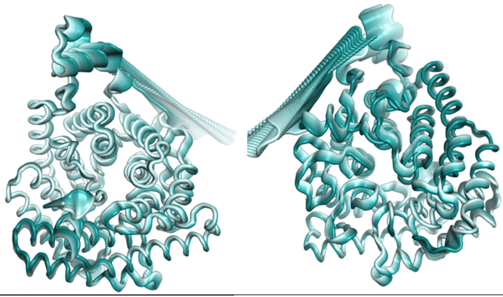	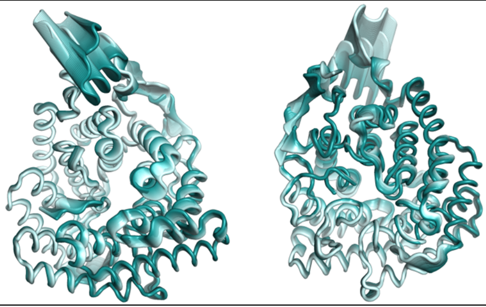
5	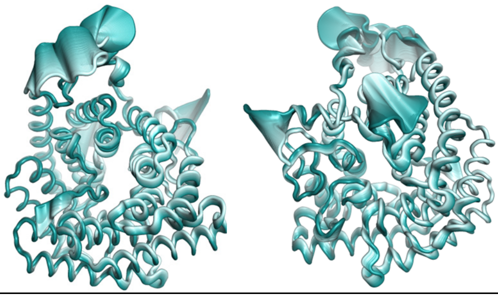	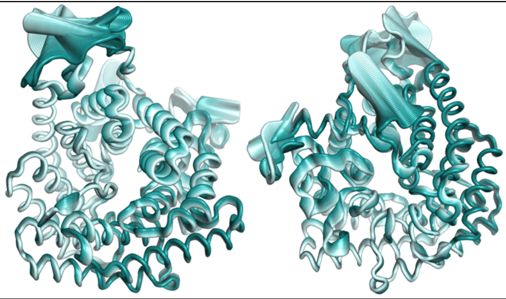

Table S14. H-bond analysis for the *R*-**ABA**:LANCL2 (AF) complex. The corresponding occupancy values are shown as percentage. Dark blue: over 50%, medium blue: from 30 to 50%, light cyan: between 20-30%, no color: absent or less than 20%. Replicas highlighted in red exhibits a ligand destabilization/unbinding (u).

**MD simulation**	**Involved residues**		
REPLICA N.	ALA 441	ARG 438	ARG 118
1	YES (92%)	YES (39%)	YES (30%)
2	YES (90%)	YES (34%)	YES (42%)
3	YES (87%)	YES (25%)	YES (69%)
4	YES (90%)	YES (42%)	YES (59%)
5	YES (91%)	YES (28%)	YES (49%)

Table 15. H-bonds analysis for the *S*-**ABA**:LANCL2 (AF) complex. The corresponding occupancy values are shown as percentage. Dark blue: over 50%, medium blue: from 30 to 50%, light cyan: between 20-30%, no color: absent or less than 20%. Replicas highlighted in red exhibits a ligand destabilization/unbinding (u).

**MD simulation**	**Involved residues**						
REPLICA N.	ALA 441	ARG 438	ARG 118	TYR 209	LYS 164	GLU 213	ARG 449
1	NO	NO	NO	YES (50%)	YES (24%)	YES (27%)	NO
2	YES (73%)	NO	YES (24%)	YES (21%)	NO	NO	YES (23%)
3	YES (94%)	NO	YES (49%)	NO	YES (37%)	YES (80%)	NO
4	YES (91%)	YES (47%)	YES (38%)	NO	YES (21%)	NO	NO
5	YES (94%)	YES (38%)	YES (53%)	NO	NO	NO	NO

Table S16. H-bonds analysis for the R-**ABA**:LANCL2 (MOE) complex. The corresponding occupancy values are shown as percentage. Dark blue: over 50%, medium blue: from 30 to 50%, light cyan: between 20-30%, no color: absent or less than 20%. Replicas highlighted in red exhibits a ligand destabilization/unbinding (u).

MD simulation	Involved residues					
REPLICA N.	ALA 441	ARG 438	ARG 118	TYR 209	ARG 449	LYS 164
1	YES (94%)	NO	YES (58%)	NO	NO	YES (25%)

2	YES (93%)	YES (40%)	YES (60%)	NO	NO	NO
3	YES (92%)	YES (36%)	YES (79%)	NO	NO	NO
4	NO	NO	NO	YES (90%)	YES (61%)	NO
5	YES (78%)	NO	YES (59%)	NO	NO	YES (24%)

Table S17. H-bonds analysis for the S-**ABA**:LANCL2 (MOE) complex. The corresponding occupancy values are shown as percentage. Dark blue: over 50%, medium blue: from 30 to 50%, light cyan: between 20-30%, no color: absent or less than 20%. Replicas highlighted in red exhibits a ligand destabilization/unbinding (u).

**MD simulation**	**Involved residues**					
REPLICA N.	ALA 441	ARG 438	ARG 118	TYR 209	ARG 449	LYS 448
1	YES (96%)	YES (28%)	YES (53%)	NO	NO	YES (24%)
2	NO	NO	NO	YES (98%)	YES (63%)	NO
**3 (u)**	NO	NO	NO	NO	NO	NO
4	NO	YES (36%)	NO	YES (47%)	NO	NO
5	NO	YES (95%)	NO	YES (98%)	YES (87%)	NO

Table S18. AR-42 docking scores as obtained via (SITE1)-IF protocol at the Alphafold (AF)-completed model (MODEL 1). The compound pose retained for the following computational analyses is highlighted in light violet.

Pose n.	docking score	glide gscore	IFDScore
1	-7.962	-7.962	-892.73
2	-7.635	-7.635	-892.69
3	-7.624	-7.624	-892.16
4	-7.073	-7.073	-891.61
5	-7.614	-7.614	-891.20
